# Thermodynamics, economy and environment analyses and optimization of series, parallel, dual-loop Kalina cycles for double-source heat recovery in cement industry

**DOI:** 10.1371/journal.pone.0315972

**Published:** 2025-02-21

**Authors:** Yali Wang, Yongjun Xu, Yongliang Qiu, Shengwang Ning

**Affiliations:** School of Electromechanical Engineering, Guangdong Polytechnic of Industry and Commerce, Guangzhou, China; GH Raisoni College of Engineering and Management Pune, INDIA

## Abstract

This research aims to investigate the heat recovery of both suspension preheater flue gas and clinker cooler hot air in cement industry. Three thermodynamic cycles including series Kalina Cycle (S-KC)、parallel Kalina Cycle (P-KC) and dual-loop Kalina Cycle (DL-KC) are introduced for converting dual-source heat resources into power to enhance the system efficiency for cement production process. Firstly, the multi-layer comprehensive evaluation models are established for the three thermodynamic cycles. Then, the parametric studies are implemented to estimate the influences of six key parameters on the system’s thermodynamic-economic-environmental performances. Meanwhile, optimization investigations consisting of thermodynamic optimal design (TOD), thermodynamic and economic optimal design (TEOD), and thermodynamic, economic and environmental optimal design (TEEOD) are considered, and the performances of systems and components are compared under three optimal design scenarios. The results prove that, for S-KC, P-KC and DL-KC, the higher net power output (*W*_*net*_) can be gained with decreasing condenser outlet temperature and regenerator temperature difference, and increasing evaporator temperature difference and superheat degree, the lower electricity production cost (*EPC*) can be acquired with decreasing condenser outlet temperature, evaporator temperature difference and regenerator temperature difference, while the less environment impact load (*EIL*) can be attained with decreasing condenser outlet temperature, regenerator temperature difference and basic ammonia concentration, and increasing superheat degree. In addition, under TOD, TEOD and TEEOD scenarios, DL-KC is the best selection from the thermodynamic, economic and environmental perspectives, with the corresponding *W*_*net*_ of 7166 kW, 6904 kW and 6838 kW, the *EPC* of 0.00476 $/kWh, 0.00369 $/kWh and 0.00362$/kWh, the *EIL* of 0.0597 mPE_China,90_/kWh, 0.0599 mPE_China,90_/kW and 0.0593 mPE_China,90_/kW. It also identifies that the evaporator unit is the key component contributing to exergy destruction and investment cost for three systems, while the pump has the maximum influence on environmental performance.

## Section 1: Introduction

Cement production is regarded as one of the most energy-intensive industry, and occupying 40%-60% of the manufacture cost is used for energy consumption in the cement plant [1]. However, a large amount of input heat energy is dissipated due to the discharges from suspension preheater flue gas, clinker cooler hot air and so on, which has been estimated up to 40% of the overall input energy [2]. Therefore, it is necessary to recover waste heat of the cement industry to reduce exhaust emission and energy consumption.

Waste heat recovery (WHR) technology has been proven to be a feasible approach for heat energy harvesting in various industries, and many researchers also consider WHR for cement production to improve energy efficiency. Wang et al. [3] considered organic Rankine cycle (ORC) for electricity generation by utilizing exhaust gas from the cement kiln cooler and evaluated the thermodynamic, economic and environmental performances of five working fluids for the combined system. Their research concluded that, for the cement plant with production of 4000 t/d, ORCs could generate electricity of 6785540 kWh/year-8121650 kWh/year, the payback period was 2.74 years to 3.42 years, and CO_2_ was reduced by 0.62%-0.74%. Utilizing the exhaust gas of rotary kiln as heat source, Fergani et al. [4] evaluated the influences of the key parameters and components on ORC’s performances by using exergy, exergoeconomic and exergoenvironmental methods. Ustaoglu et al. [5] had also designed an ORC for the exhausted gas of rotary kiln with heat energy of 30.5MW. They concluded that Isentropic and R245fa exhibited the best thermal and exergetic performances. In addition, heat exchanger and evaporator had higher exergy destruction, accounting for 80% of the total exergy destruction. Adopting actual data from cement plant, Ahmed et al. [6] proposed the ORC for recovering waste heat from kiln exhaust gas, and examined the system performances by employing R134a as working fluid. Their findings shown that the proposed system could obtain 1 MW power, and ORC had better performance than steam circle from the exergy perspective. Jamali et al. [7] had studied a novel solar-ORC combined system installing on a cement plant. Research investigation was conducted from the energetic, exergetic and thermoeconomic perspectives. They reported that 17.4MW and 18.4MW electricity could be generated by this system in summer and winter, respectively. Moreover, the optimized results revealed that, compared with winter, the system had higher product cost rate by 0.47% but with higher exergy efficiency by 39% in summer. Júnior et al. [8] considered Kalina cycle (KC) harvesting the flue gas of the cyclone preheater in cement plant with an annual capacity of 2100 tons. The outcomes showed that the increasing ammonia concentration and the declining pinch point of the evaporator resulted in an increase of net power, while the increasing pressure of the turbine reduced the investment cost. In addition, boiler and the condenser were proved to be the critical components in terms of cost estimation. Nami et al. [2] considered the heat source of cement plant with the discharge temperature of 523 K and volumetric flow of 18.43 kg/s for WHR. They compared RC-CCHP and ORC-CCHP by using energy, exergy and exergoeconomic techniques. It was shown that ORC-CCHP had better performance in thermodynamics, while RC-CCHP had more advantage in economics. KCS1 and KCS34 as bottoming circles for WHR in cement industry were analyzed and compared considering the power ‌generation and system cost by Horta et al. [9]. They concluded that KCS1 was the better alternative for greater capacity and medium-high temperature heat source according to the generated net power, while KCS34 was more promising for smaller capacity and low temperature heat source from the economic perspective. Mahmoud et al. [10] explored separate and combined integration of KC for WHR in a cement factory. Their research determined the design parameters for KCs and concluded that the combined system had higher power output and cost saving. Bisulandu et al. [11] adopted three novel configurations of KC system to recover waste heat from cement kiln, and identified the most effective WHR system based on thermodynamic comparison. Laazaar et al. [12] had studied Stirling engine for electricity production by recovering waste heat of cooling clinker in cement production. The influences of key parameters on the output power and efficiency of Stirling engine were investigated. Furthermore, the optimization regarding Stirling engine specification was conducted for acquiring the best design to improve the thermodynamic performance. The Analytic Hierarchy Process methodology was adopted to evaluate the selection scheme of WHR in the cement industry with a daily production capacity of 5000 tons by Marenco-Porto et al. [13]. In their research, four criteria and five scenarios were examined for energy-economic-environmental performance of ORC, RORC and TLC. The results showed that RORC was superior to the other two cycles due to the higher output power, lower emission reduction and net present value.

According to the literature review, many researches have carried out WHR for cement industry, and investigated system performances from the perspectives of thermodynamics or/and economics, and even CO_2_ emission reduction. Currently, however, their research consider only the preheater exhaust energy into account. In fact, although the hot air from the clinker cooler has a lower temperature, it still contains considerable heat energy due to its large mass flow rate. Limited studies have simultaneously considered the heat recovery of both preheater exhaust gas and clinker cooler hot air in cement production process. Rad et al. [1] considered Rankine cycle as bottom cycle to harvest the heat energy of hot exhaust gas from kiln chimney and clinker cooler. They investigated the optimal pressure for the recovery system by employing energy and exergy methods. Furthermore, the impacts of the key parameters on the optimum pressure were explored. Karellas et al. [14] also considered double-source heat recovery for cement industry, involving exhaust gas and hot air with temperatures of 380°C and 360°C, respectively. Their study had analyzed and compared the water-steam RC and ORC based on energy and exergy approaches. They reported that when the exhaust gas temperature exceeded 310°C, the water-steam cycle was more favorable than ORC. The discharged gases of grate cooler and preheater were also utilized to drive the steam RC for electrical generator by Ghalandari et al. [15]. In their research, 5.2MWh electrical energy could generate by the system and the capital return time was evaluated to be 6.7 years. Moreira et al. [16] had evaluated simple and regenerative ORCs driven by dual heat source in cement plants with daily production capacities from 3000 ton to 6300 ton. Their work shown that R141b, R11, and R123 were more competitive in technical properties. For the proposed cycles, ORCs could produce power reaching 4000 kW to 9000 kW, while specific generation costs ranging from 0.09 R$/kWh-0.11 R$/kWh. Heat recovery from grate cooler and suspension preheater were also conducted by Sanaye et al. [17]. The RC and ORC were modelled and optimized based on thermo-economic and environmental analysis. The results shown that the system adopted water and toluene as working fluid could produce power production of 9.14 MW and 6.56 MW, cost saving of 2.1×106 $/year and 1.46 × 106 $/year, and CO_2_ emission reduction of 5.36×10^4^ ton/year and 3.81×10^4^ ton/year, respectively. Chen et al. [18] considered a novel WHR system that integrated the discharged gas of cyclone preheater and clinker cooler with a coal-fired power plant. Their project was investigated from the perspectives of thermodynamics and economics. Compared to the conventional system, the integrated design could improve power efficiency and net efficiency by 18.11% and 8.11%, while decline the payback period by 2.03 years.

As can be inferred, the related researches involving WHR of dual heat source in cement industry mainly focus on ORC. In fact, KC is another potential technology in competition with ORC for WHR, and it has been applied in cogeneration, geotherm, diesel engine, gas turbine and so on, which are summarized and compared in Table 1. It is noted that KC is not unexplored for WHR of double-source in cement manufacture. Considering that, especially some researches indicate that the KC exhibits better thermodynamic performance [36, 37], and lower specific investment cost [38, 39], we concentrate on this method for double-source heat recovery in cement industry. In addition, working fluid selection, configuration comparison and performance optimization in the existing research involving cement’s WHR are usually evaluated based on thermodynamic or/and economic viewpoints, only the study [16] has estimated the environmental performance but simply involving CO_2_. In other words, the comprehensive investigation considering thermodynamic, economic, and environmental performances is not well studied, especially environmental research based on life cycle assessment (LCA), which is to evaluate the environmental potential impacts generated by the whole life-span of construction phase, operation phase, and decommissioning phase, has not been well addressed.

**Table 1:** Application and comparison of KC for WHR in different scenarios.

Application	System configuration	Analysis			Reference
		Thermodynamics	Economics	Environment	
Cogeneration	KCS34	√	√	√ (CO_2_ emission reduction)	[19]
	Modified KCS	√	√	×	[20]
	Modified KCS	√	√	×	[21]
Geotherm	KCS11, KCS34, KCS34g	√	×	×	[22]
	KCS34	√	√	×	[23]
	KCS34	√	×	×	[24]
Diesel engine	Modified KCS	√	√	√ (CO_2_ emission reduction)	[25]
	Modified KCS	√	×	×	[26]
	Modified KCS	√	×	×	[27]
Gas turbine	KCS-11	√	√	√ (NO_x_ and CO)	[28]
	Modified KCS	√	×	×	[29]
	Modified KCS	√	√	×	[30]
Solar thermal power plant	Modified KCS	√	√	√ (ecological performance)	[31]
	KCS12, KCS123, KCS234, KCS1234	√	×	×	[32]
Iron and steel industry	KCS-11	√	√	×	[33]
Coal-fired power plant	KCS 11	√	√	×	[34]
Solid waste power plant	KCS34	√	√	×	[35]

Therefore, this paper proposes three novel configurations of KC including series、parallel、dual-loop KCs for double-source WHR of both exhaust gas from suspension preheater and hot air from clinker cooler in cement plant to improve the system energy efficiency. Investigation and comparison of the designed systems are carried out in combination of thermodynamic, economic and environmental factors. The article is arranged as follows. Firstly, three proposed KCs for double-source heat utilization in cement industry are introduced. Then, the multi-layer comprehensive evaluation including thermodynamic model based on the first and second laws of thermodynamics, economic model based on module cost, and environmental model based on LCA are established. Afterwards, the influences of key operating parameters on thermodynamic, economic and environmental performances are investigated. Finally, single-, bi- and tri-objective optimization are carried out using genetic algorithm method, and further investigation is conducted on the thermo-economic-environmental comparisons of system and component under three optimal design scenarios.

The novelty of this study lies in the following points: (i) the KC for double-source WHR in cement plant is studied for the first time; (ii) performance investigation of the designed systems is analyzed and compared from thermodynamic and economic aspects, as well as from environmental perspective based on LCA; and (iii) the optimization research considers three design scenarios and distinguishes the most suitable system based on different criteria for each scenario.

## Section 2: Methodology

**Fig 1:**
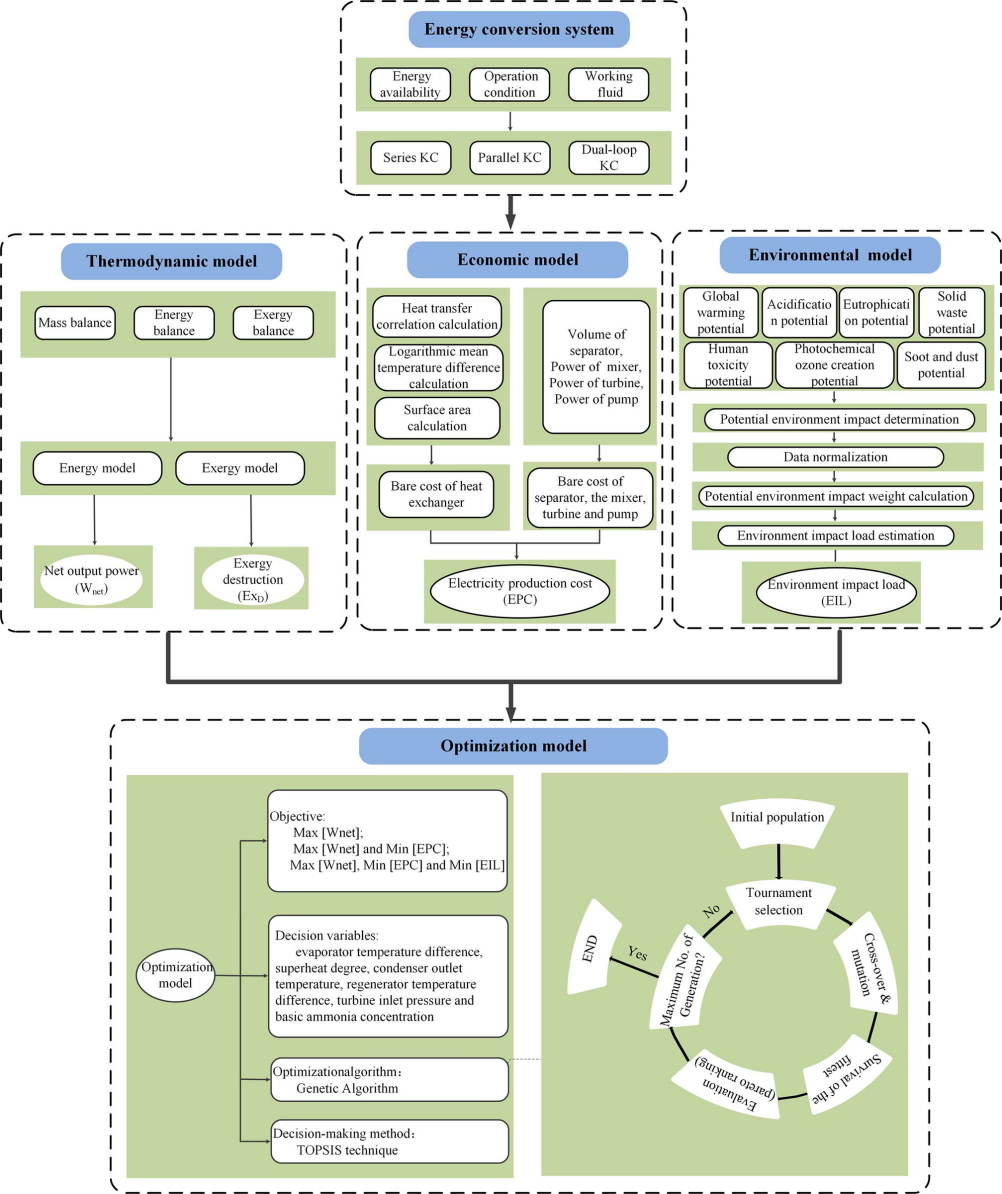
The general flowchart of research methodology.

Fig 1 displays the general flowchart of the research methodology in this paper. The subsection 2.1 introduces the three proposed configurations of KC system. The subsection 2.2 describes the energy availability in cement industry for WHR. The subsection 2.3–2.5 concentrates on the modelling method including thermodynamic model, economic model and environmental model. The subsection 2.6 presents the optimization methodology involving the objective function, decision variable and Pareto-optimal solution decision making method.

### 2.1. KCs description

**Fig 2:**
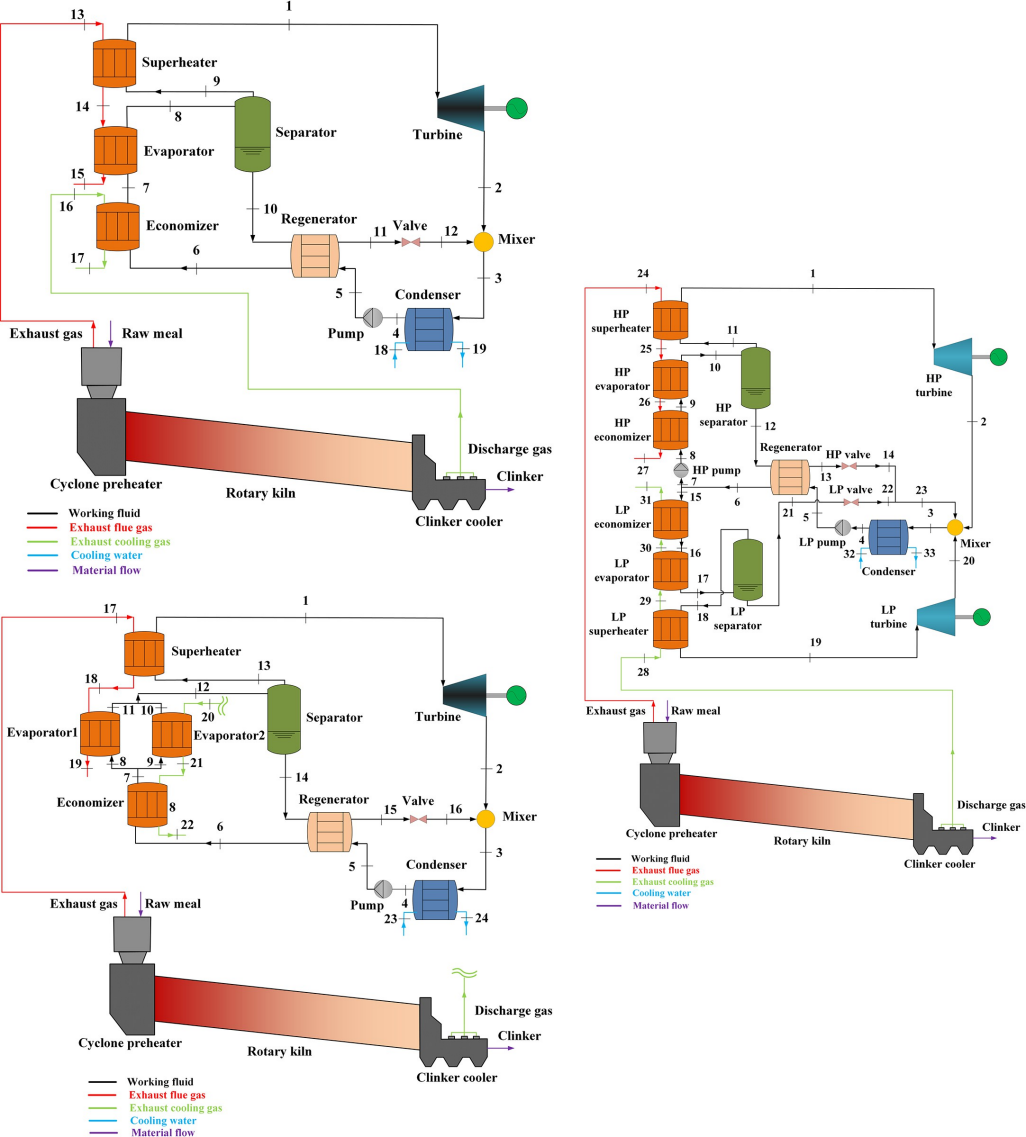
System schematic diagrams for WHR in cement industry: (a) S-KC, (b) P-KC and (c) DL-KC.

In this study, the KC is utilized to recover the waste heat of suspension preheater and clinker cooler in the cement industry. Fig 2 presents the schematic diagrams of series KC (S-KC)、parallel KC (P-KC)、dual-loop KC (DL-KC). In Fig 2(A), the S-KC is consisted of a turbine, a condenser, a pump, a regenerator, a separator, a mixer, a throttle valve, an evaporator unit which is composed of a superheater, an evaporator and an economizer. In Fig 2(B), the composition of P-KC is similar to the S-KC, except that the evaporator unit include two evaporators. In Fig 2(C), the DL-KC involves a condenser, a regenerator, a mixer, two turbines, two pumps, two separators, two throttle valves, two evaporator units. And the evaporator unit also includes a superheater, an evaporator and an economizer.

The S-KC is presented in Fig 2(A). The basic ammonia-water mixture is preheated in the economizer, turns to two-phase mixture in the evaporator, and then is split to ammonia-rich vapor and ammonia-poor solution by the separator. The ammonia-rich vapor is superheated in the superheater and then flows to the turbine where the fluid is expanded to turn energy into power. The ammonia-poor solution enters the regenerator, depressurized by the throttle valve, and then combines with the turbine’s discharge to produce the basic ammonia-water mixture in the mixer. After that, the basic ammonia-water mixture is cooled by cooling water in the condenser, after being elevated its pressure by the pump, flows through the regenerator to recover heat, and finally delivered to the economizer recommencing the cycle again. The P-KC is displayed in Fig 2(B). Two parallel evaporators are arranged in the evaporation unit. Unlike S-KC, after being heated in the economizer, the basic ammonia-water mixture is divided to two liquid streams, flowing to evaporator 1 and evaporator 2, where the working fluids are vaporized to two-phase mixture. The steam flows exiting the two evaporators are mixed and then delivered to the separator. The DL-KC is presented in Fig 2(C). This configuration is comprised of a high-pressure (HP) loop and a low-pressure (LP) loop. Two pumps are arranged to adjust different pressure levels in the system. As shown, the basic ammonia-water mixture exhausted from the regenerator is split into two streams, one is for the HP loop and the other is for the LP loop. As for the HP loop, the working fluid orderly passes through the HP pump, HP economizer, HP evaporator and HP separator. The ammonia-rich vapor from the top of HP separator enters the HP turbine for power production, and the ammonia-poor solution from the bottom of HP separator is sent to the regenerator and HP throttle valve. As for the LP loop, the basic ammonia-water mixture works in a similar manner to HP loop, but without a regenerator. The combined emissions of HP and LP throttle valve are mixed with the exhaust steams from HP and LP turbines to form basic ammonia-water mixture in the mixer, then go through the condenser, LP pump and regenerator sequentially.

### 2.2. Energy availability

In the present study, the original input data for dual heat sources recovery in cement production are obtained from the reference [16]. The available energy is sourced from the heat energy of hot air from clinker cooler and exhaust gas from suspension preheater of a Brazil cement plant with a daily production capacity of 3500 ton. The gas information involving inlet temperature, outlet temperature and mass flow are shown in Table 2. It is worth noting that the values relating to hot air from the clinker cooler correspond to state 13, 14 and 15 in Fig 2(A), 17, 18 and 19 in Fig 2(B), 24, 25, 26 and 27 in Fig 2(C) for S-KC, P-KC and DL-KC, respectively. The values relating to exhaust gas from the suspension preheater correspond to state 16 and 17 in Fig 2(A), 20, 21 and 22 in Fig 2(B), 28, 29, 30 and 31 in Fig 2(C) for the S-KC, P-KC and DL-KC, respectively.

**Table 2:** Energy availability data for WHR in cement production.

Parameter	Unit	Value	Parameter	Unit	Value
Hot air from clinker cooler			Exhaust gas from suspension preheater		
Inlet temperature	K	713	Inlet temperature	K	583
Outlet temperature	K	387	Outlet temperature	K	501
Mass flow	kg/s	48.15	Mass flow	kg/s	88.03

Note that the temperature unit in reference [16] is °C, and we have converted it to K in Table 2.

### 2.3. Thermodynamic model

In the current research, the whole numerical model of KC is developed in the MATLAB (versionR2016a) software and thermo-physical properties of ammonia-water mixture are acquired by REFPROP (version9.1) database. In addition, the following hypotheses are specified to facilitate the analysis: (1) the cycle runs under a steady condition; (2) the variations in kinetic and potential energy are disregarded; (3) the heat and friction losses are negligible; (4) the pressure drops are ignored. The working conditions of the systems are shown in Table 3.

**Table 3:** Working conditions for the designed KCs.

Parameter	Unit	Value	Circle
Turbine isentropic efficiency	%	80	S-KC, P-KC, DL-KC
Turbine mechanical efficiency	%	90	S-KC, P-KC, DL-KC
Pump isentropic efficiency	%	80	S-KC, P-KC, DL-KC
Pump mechanical efficiency	%	90	S-KC, P-KC, DL-KC
Temperature difference of evaporator	K	8	S-KC, P-KC, DL-KC
Degree of superheat	K	40	S-KC, P-KC, DL-KC
Outlet temperature of condenser	K	300	S-KC, P-KC, DL-KC
Temperature difference of regenerator	K	26	S-KC, P-KC, DL-KC
Pinch point in the evaporator	K	180	P-KC, DL-KC
The pressure of the turbine inlet (HP turbine for DL-KC)	kPa	5500	S-KC, P-KC, DL-KC
The pressure of the LP turbine inlet	kPa	2500	DL-KC
Cooling water inlet temperature	K	285	S-KC, P-KC, DL-KC
Cooling water outlet temperature	K	305	S-KC, P-KC, DL-KC
Basic ammonia concentration	%	75	S-KC, P-KC, DL-KC
Environmental temperature	K	293	S-KC, P-KC, DL-KC

The thermodynamic models of S-KC, P-KC and DL-KC for each equipment are established based on mass, energy and exergy balances. Eq (1) and Eq (2) give the mass and concentration conservation of each material, where *x* in Eq (2) refers to mass concentration of NH_3_ in the solution. Eq (3) shows the energy equation for a control volume. Eq (4) calculates the exergy of the state point, where *h* and *s* refer to the specific enthalpy and specific entropy; *h*_0_ and *s*_0_ are the properties in the dead state at T_0_ and P_0_. Eq (5) calculates the exergy destruction of a component. Eq (6) gives the total exergy destruction of a system.

$$\sum m_{in}=\sum m_{out}$$ (1)

$$\sum {\left(mx\right)}_{in}=\sum {\left(mx\right)}_{out}$$ (2)

$$Q+\sum m_{in}h_{in}=W+\sum m_{out}h_{out}$$ (3)

$$Ex=m[\left(h-h_{0})-T_{0}(s-s_{0}\right)]$$ (4)

$$Ex_{D}=\sum Ex_{in}-\sum Ex_{out}-W$$ (5)

$$\sum Ex_{D,tot}=\sum Ex_{D}$$ (6)

The net output power (*W*_*net*_) produced by a system is determined by Eq (7), where *W*_*turb*_ is the turbine’s power production, and *W*_*p*_ is the pump’s power consumption.


$$W_{net}=W_{turb}-W_{p}$$ (7)

### 2.4. Economic model

#### 2.4.1. Transfer surface area of the heat exchanger

To evaluate the economic performance of a system, the surface area calculation of the heat exchanger needs to be carried out firstly. In this study, the finned-tube heat exchanger is considered for the evaporator unit, while the shell-tube heat exchanger is selected for condenser and regenerator. Eq (8) calculates the transfer surface area (A). Eq (9) formulates the logarithmic mean temperature difference (Δ*T*_*m*_) presented in reference [40], where *ΔT*_*max*_ and *ΔT*_*min*_ mean the maximum and minimum values of temperature differences at the terminal of the heat exchanger. Eq (10) refers to the overall heat transfer coefficient provided in reference [41], where *a*_*hs*_ and *a*_*cs*_ indicate the heat transfer coefficients of hot-side and cold-side of heat exchanger, *t* and *λ* are the thickness and thermal conductivity of a substance.

$$Q=UA\Delta T_{m}$$ (8)

$$\Delta T_{m}=({\Delta T}_{max}-{\Delta T}_{min})/In({\Delta T}_{max}/{\Delta T}_{min})$$ (9)

$$\frac{1}{U}=\frac{1}{a_{hs}}+\frac{t}{\lambda }+\frac{1}{a_{cs}}$$ (10)

The calculation equation related to heat transfer correlation of heat exchanger is based on flow passage type and working fluid state. Eq (11) and Eq (12) calculate the heat transfer coefficient of the single-phase flow for evaporation unit and the cold side for regenerator [42]. Eq (13) gives the heat transfer coefficient of the two-phase flow for evaporation unit [43]. Eq (14) shows the correlation of heat transfer coefficient between heat source and evaporation unit [44]. Eq (15) gives the heat transfer coefficient of the single-phase flow for cooling process and the hot side for regenerator [45]. Eq (16) calculates the heat transfer coefficient of the two-phase for condensation process [46]. Eq (17) formulates the heat transfer coefficient of cooling water side [42].


ai=λiDi((fi/8)ReiPri12.7(fi/8)0.5(Pri2/3−1)+1.07) (11)

fi=1(1.82log10Rei−1.64)2 (12)

$$\alpha_{i}=\alpha_{slp}{\left\{{\left[(1-x_{i})+1.2{x_{i}}^{0.4}(1-x_{i})(\frac{\rho_{slp}}{\rho_{svp}}{)}^{0.37}\right]}^{-2.2}+{\left[\frac{\alpha_{svp}}{\alpha_{slp}}{x_{i}}^{0.01}(1+8(1-x_{i}{)}^{0.7})(\frac{\rho_{slp}}{\rho_{svp}}{)}^{0.67}\right]}^{-2}\right\}}^{-0.5}$$ (13)

αi=0.35Rei0.6Pri0.3 (14)

$$\alpha_{i}=\left\{ \begin{array}{c} 0.51{Re}_{i}^{0.5}{Pr}_{i}^{0.36}\;(40<{Re}_{i}\leq 1000)\\ 0.26{Re}_{i}^{0.6}{Pr}_{i}^{0.36}\;(1000<{Re}_{i}\leq 2\times 10^{5}) \end{array}\right.$$ (15)

$$\alpha_{i}=\alpha_{slp}\left[(1-x_{i}{)}^{0.8}+\frac{3.8x^{0.76}(1-x_{i}{)}^{0.04}}{(\frac{p}{p_{c}}{)}^{0.38}}\right]$$ (16)

$$\alpha_{i}=0.023{Re}_{i}^{0.8}{Pr}_{i}^{0.4}$$ (17)

#### 2.4.2. Cost model

The module costing technique presented in reference [47] is employed to estimate the investment cost for the proposal systems. Eqs (18)–(21) are used to calculate each component’s bare cost [48], where P is design pressure of the component and X represents size parameter according with the heat exchanger’s heat transfer area, the separator’s volume, the mixer, turbine and pump’s power. The correlation coefficients for component cost evaluation are listed in Table 4.


$$C_{bm}=C_{p}F_{bm}$$ (18)

$$\mathrm{lg}\;C_{p}=K_{1}+K_{2}\;\mathrm{lg}\;X+K_{3}(\mathrm{lg}\;X{)}^{2}$$ (19)

$$F_{bm}=B_{1}+B_{2}F_{M}F_{P}$$ (20)

$$\mathrm{lg}\;F_{p}=C_{1}+C_{2}\;\mathrm{lg}\;P+C_{3}(\mathrm{lg}\;P{)}^{2}$$ (21)

**Table 4:** Correlation coefficient of the component cost evaluation [49].

Item	Turbine	Pump	Evaporator	Condenser	Regenerator	Separator	Mixer
*K*_1_	2.7051	3.8696	3.9912	4.3247	4.3247	3.4974	3.4092
*K*_2_	1.4398	0.3161	0.0668	-0.303	-0.303	0.4485	0.4896
*K*_3_	-0.1776	0.1220	0.2430	0.1634	0.1634	0.1074	0.0030
*C*_1_	/	-0.2454	/	/	/	/	/
*C*_2_	/	0.2590	/	/	/	/	/
*C*_3_	/	-0.0136	/	/	/	/	/
*B*_1_	/	1.89	1.74	1.63	1.63	2.25	/
*B*_2_	/	1.35	1.55	1.66	1.66	1.82	/
*F*_*M*_	/	2.35	1.8	1.38	1.38	3.2	/
*F*_*bm*_	6.2	/	/	/	/	/	1.38

The electricity production cost (*EPC*) means the cost to 1 kWh *W*_*net*_ created by a system, which is defined as the evaluation criterion for economic performance. Eq (22) gives the cost of all components in 2001. Eq (23) gives the actual cost of the system in 2022, where *CEPCI*2001 and *CEPCI*2022 are set to 397 and 816.3 [50, 51], respectively. Eq (24) shows the capital recovery factor, the *i* and *time* are the interest rate and life span, assuming to 5% and 16 years. Eq (25) calculates *EPC*, where *f*_*k*_ indicates the cost coefficient of the operation, upkeep and insurance, and its value is set to 1.65%; *h*_*full−load*_ is the full-load working hour, and its value is specified as 7500h.


$$Cost2001=\sum C_{bm}$$ (22)

Cost2022=Cost2001⋅CEPCI2022/CEPCI2001 (23)

$$CRF=i\cdot (1+i{)}^{time}/((1+i{)}^{time}-1)$$ (24)

$$EPC=(Cost2022\cdot CRF+f_{k}\cdot Cost2022)/(W_{net}\cdot h_{\mathrm{full}-\mathrm{load}})$$ (25)

### 2.5. Environmental model

**Fig 3:**
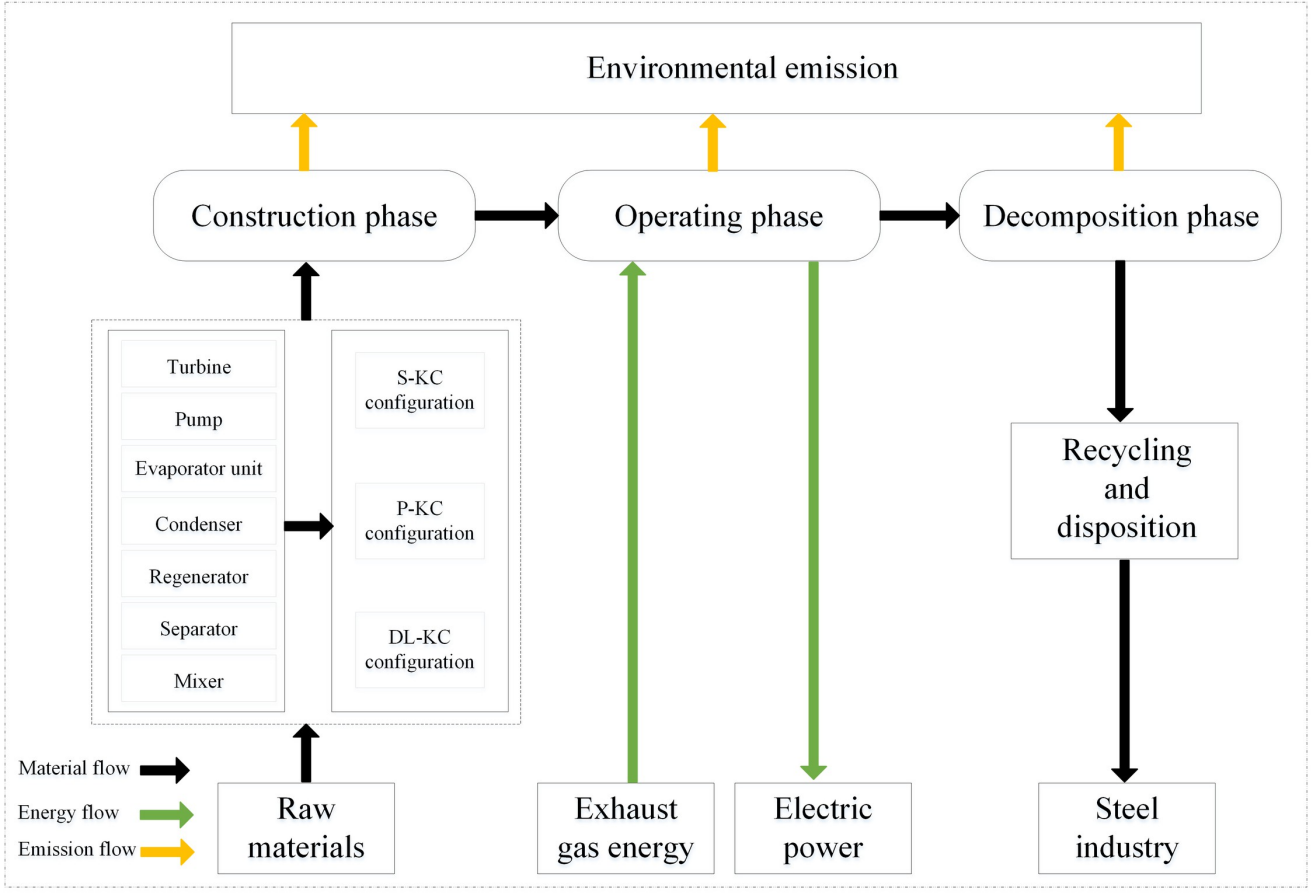
Life-cycle boundary of the thermodynamic cycle.

The LCA technique is employed to evaluate the environmental performance of the studied systems. The principles of LCA comply with ISO14040 criterion. LCA involves the whole life-span pollutant emission covering the construction period, operation period and decommissioning period, and the life-cycle boundary of the system is displayed in Fig 3. In the current research, GWP (global warming potential), AP (acidification potential), EP (eutrophication potential), HTP (human toxicity potential), POCP (photochemical ozone creation potential), SWP (solid waste potential) and SAP (soot and dust potential) are considered for the environment impact potentials of the system. The various environmental impact potentials have different influences on the environmental performance, and the equivalency coefficients, standardization benchmarks and weighting coefficients listed in Table 5 are introduced to reflect their influences extent on the environment. Furthermore, the basic pollutant exhausts data of steel manufacture, power production and lorry transportation are shown in Table 6.

**Table 5:** Environment impact type, equivalency coefficient, standardization benchmark and weighting coefficient [3].

Type	Pollutant exhaust	Equivalency coefficient	Standardization benchmark	Weighting coefficient
GWP	CO_2_	1	8700 kg CO_2_ eq/(p▪a)	0.83
	CH_4_	25		
	NO_x_	320		
	CO	2		
AP	SO_2_	1	36 kg SO_2_ eq/(p▪a)	0.73
	NO_x_	0.7		
	H_2_S	1.88		
	HCl	0.88		
EP	NO_x_	1.35	62 kg NO_3_ eq/(p▪a)	0.73
	CH_4_	3.44		
	NO_3_	1		
HTP	CO	0.012	24.65 kg/(p▪a)	0.73
	SO_2_	1.2		
	NO_x_	0.78		
POCP	HC (hydrocarbon)	0.398	0.65 kg C_2_H_4_ eq/(p▪a)	0.53
SWP	Solid waste	1	251 kg/(p▪a)	0.62
SAP	Soot and dust	1	18 kg/(p▪a)	0.61

**Table 6:** Pollutant exhaust of the per kg of steel manufacture, per kWh of power production and per tn·km of lorry transportation [52].

Exhaust (g)	CO_2_	CH_4_	NO_x_	CO	HC	SO_2_	H_2_S	HCl	SW	SA
Steel (kg^-1^)	410	0.9	0.8	5.5	0.095	2.55	0.00435	0.0437	243	15
Power (kWh^-1^)	1141.2	/	5.2164	2.772	/	10.314	/	/	45.792	9.5472
Lorry transportation ((tn•km) ^-1^)	23.77	/	0.7641	2.758	0.3416	0.182	/	/	/	/

The establishment of environment model consists of potential environment impact determination, data normalization, potential environment impact weight calculation and environment impact load estimation [3]. Eq (26) gives the system’s life-span contribution on the potential environment impact *j*, where *EP* (*j*)_*i*_ is the pollutant exhaust contribution of *i* to the environment impact potential of *j*,*Q*(*j*)_*i*_ means the pollutant exhaust amount of *i* and *EF*(*j*)_*i*_ indicates the pollutant exhaust equivalent coefficient of *i* to the environment impact potential of *j*. Eq (27) is employed to standardize environment impact potential of *j*, where *ER*(*j*)_90_ is the entire environment impact potential of *j* for China in 1990. Eqs (28)–(29) calculate the potential environment impact weight, *WF*(*j*) is the environment impact potential weighting factor of *j* and *ER*(*j*)_2000_ is the entire environment impact potential of *j* for China in 2000. Eq (30) formulates the environment impact load (*EIL*).


$$EP\;(j)=\sum EP\;(j{)}_{i}=\sum \;[Q\;(j{)}_{i}\times EF\;(j{)}_{i}]$$ (26)

$$NEP\;(j)=EP\;(j)/ER\;(j{)}_{90}$$ (27)

WP(j)=NEP(j)WF(j) (28)

$$WF(j)=ER\;(j{)}_{90}/ER\;(j{)}_{2000}$$ (29)

EIL=∑WP(j) (30)

### 2.6. Optimization model

In the present study, single- and multi-objective optimization are carried out for S-KC, P-KC and DL-KC. The single-objective optimization aims at maximizing *W*_*net*_, and Eq (31) is adopted as the optimization model. The multi-objective optimization involves the bi-objective optimization with maximizing *W*_*net*_ and minimizing *EPC*, as well as the tri-objective optimization with maximizing *W*_*net*_, minimizing *EPC* and minimizing *EIL*. Eq (32) is employed as the mathematical model for bi-objective optimization, while Eq (33) is chosen as the mathematical model for tri-objective optimization. The decision variables include evaporator temperature difference (*ΔT*_*eva*_), superheat degree (*ΔT*_*sup*_), condenser outlet temperature (*T*_*con*_), regenerator temperature difference (Δ*T*_*reg*_), turbine inlet pressure (*P*_1_) and basic ammonia concentration (*x*).

The optimization models corresponding to the single-, bi- and tri-objective optimization are presented as follows:

$$\left\{ \begin{array}{c} max\;W_{net}\\ Subject\;to:\\ 5\leq {\Delta T}_{eva}\;(K)\leq 20\\ 25\leq \Delta T_{sup}\;(K)\leq 40\\ 290\leq T_{con}\leq 300\\ 13\leq {\Delta T}_{reg}\leq 26\\ 3000\leq P_{1}\leq 5500\\ 0.73\leq x\leq 0.85 \end{array}\right.$$ (31)

$$\left\{ \begin{array}{c} max\;W_{net}\;and\;min\;EPC\\ Subject\;to:\\ 5\leq {\Delta T}_{eva}\;(K)\leq 20\\ 25\leq \Delta T_{sup}\;(K)\leq 40\\ 290\leq T_{con}\leq 300\\ 13\leq {\Delta T}_{reg}\leq 26\\ 3000\leq P_{1}\leq 5500\\ 0.73\leq x\leq 0.85 \end{array}\right.$$ (32)

$$\left\{ \begin{array}{c} max\;W_{net},\;min\;EPC\;and\;min\;EIL\\ Subject\;to:\\ 5\leq {\Delta T}_{eva}\;(K)\leq 20\\ 25\leq \Delta T_{sup}\;(K)\leq 40\\ 290\leq T_{con}\leq 300\\ 13\leq {\Delta T}_{reg}\leq 26\\ 3000\leq P_{1}\leq 5500\\ 0.73\leq x\leq 0.85 \end{array}\right.$$ (33)

Genetic Algorithm procedure is used to perform single- and multi-objective optimization for the proposed KCs in the MATLAB. This approach is also selected in this study because it is an approximate solution for finding optimal values referring to multiple variables and non-linear or discrete functions, as demonstrated in reference [53]. The input parameters for genetic algorithm optimization are as follows: population size of 200, maximum generation of 500, crossover fraction of 0.8 and mutation fraction of 0.2. In addition, it is worth mentioning that for multi-objective optimization, the result is a series of optimal solutions, widely known as Pareto frontier solution, and each point of the Pareto frontier solution could be considered as the desired optimal solution. The point that is trade-off solution for conflicting optimization objectives, is defined as the Pareto-optimal solution. Usually, the Pareto-optimal solution choosing from the Pareto frontier requires decision making. TOPSIS technique given by Hwang [54], is employed to specify the Pareto-optimal solution for multi-objective study.

## Section 3: Results and discussions

### 3.1. Parametric analyses

**Fig 4:**
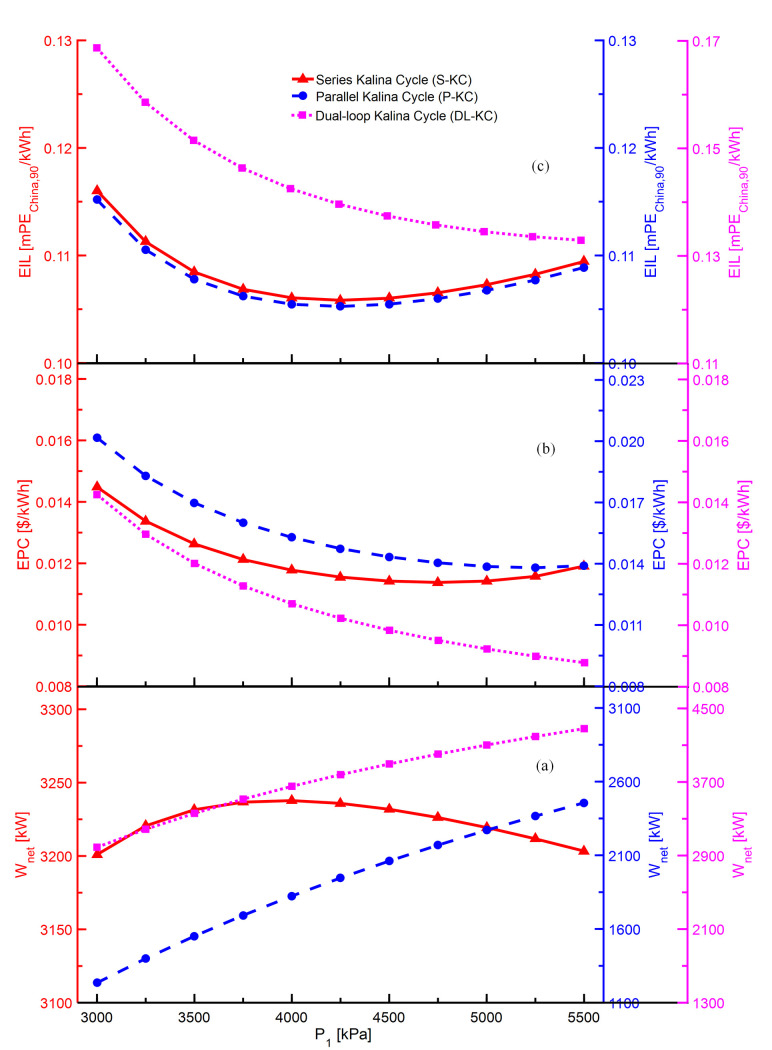
Performance variations of S-KC, P-KC and DL-KC with *P*_1_: (a) *W*_*net*_, (b) *EPC* and (c) *EIL*.

The variations of *W*_*net*_,*EPC* and *EIL* with *P*_1_ for S-KC, P-KC and DL-KC are shown in Fig 4. Referring to Fig 4(A), when the *P*_1_ varies from 3000 kPa to 5500 kPa, the *W*_*net*_ of S-KC firstly raises and then decreases, while the *W*_*net*_ of both P-KC and DL-KC show the increasing trend. Regarding DL-KC, due to the existence of dual-loop circle, its *W*_*net*_ is determined by the combined action of the HP and LP turbines. In this case, the power generated by the LP turbine remains unchanged, which means that the *P*_1_ has no influence on it. The turbine’s power generation is comprehensively determined by the mass flow of ammonia-rich vapor and enthalpy difference. For S-KC, P-KC and HP loop of DL-KC, enthalpy difference in turbine increases with the increasing *P*_1_, but the mass flow’s variation of ammonia-rich vapor is related to system configuration. As the *P*_1_ increases to 5500 kPa, ammonia-rich vapor mass flow varies from 29.3 kg/s to 18.2 kg/s for S-KC, from 11.7 kg/s to 14.4 kg/s for P-KC and from 13.5 kg/s to 12.6 kg/s for DL-KC. Regarding S-KC, the increase in enthalpy difference of turbine is predominant first, so the *W*_*net*_ increases, and later the decrease in mass flow of ammonia-rich vapor is superior, thus the *W*_*net*_ decreases. Regarding P-KC, the mass flow of ammonia-rich vapor and enthalpy difference in the turbine both raise, which lead to the growth in *W*_*net*_. Regarding DL-KC, despite the mass flow of ammonia-rich vapor declines, the increase in enthalpy difference of the turbine has a greater influence, hence *W*_*net*_ still shows an increasing trend. As observed, the conclusion is distinct in different studies. The trend of *W*_*net*_ is similar to the S-KC in the research [55], while that remains decreasing with the increasing turbine inlet pressure in the literature [35]. This could be the fact that the turbine inlet pressure has different effects on the mass flow of working fluid due to different configurations of KC system. As shown in Fig 4(B), the *EPC* of S-KC, P-KC shows a raising trend after experiencing a reduction, while the *EPC* of DL-KC keeps declining. *EPC* relies on the system investment cost and *W*_*net*_, as demonstrated in Eq (25), it is positively correlated with the system investment cost, but negatively correlated with the *W*_*net*_. For S-KC, with the increase of *P*_1_, the system investment cost decreases and the *W*_*net*_ rises, which cause a decrease in *EPC* at first. With the further increase of *P*_1_, the system investment cost grows and *W*_*net*_ decreases, thus the *EPC* increases afterward. For P-KC, the increasing *P*_1_ leads to the growth in both system investment cost and *W*_*net*_, and the influence of the growing *W*_*net*_ outweighs that of the increasing system investment cost, hence inducing a decrease in *EPC* as the *P*_1_ changing from 3000 kPa to 5250 kPa. It is noted that when the *P*_1_ is close to 5500 kPa, the increasing system investment cost becomes gradually obvious, and then the *EPC* shows a slightly increasing trend. For DL-KC, an increase in *P*_1_ enables lower system cost and higher *W*_*net*_, which causes the reduction of *EPC*. As displayed in Fig 4(C), the *EIL* of S-KC and P-KC first decrease and then increase, while that of DL-KC keeps dropping with the variation of *P*_1_. For S-KC, the decline in mass flow of the basic ammonia-water mixture and the increase in enthalpy difference of the pump cause system’s power consumption to decrease first and then increase with the increasing *P*_1_, then leading to a similar manner for system emissions. When the *P*_1_ is at a lower value, the decreasing emissions and the rising *W*_*net*_ lead to a decrease in *EIL*, while the *P*_1_ is at a higher value, the rising emissions and the decreasing *W*_*net*_ lead to an increase in *EIL*. For P-KC, with the increasing *P*_1_, the system emissions keep rising because of the increasing power consumption of the pump. The effect of the rising *W*_*net*_ exceeds that of system emissions, leading to the decline in *EIL* at first, and then the system emissions increase faster than that of *W*_*net*_, resulting in the increment in *EIL*. For DL-KC, with the increase of *P*_1_, although the LP pump’s power consumption decreases, HP pump’s power consumption shows a greater advantageous in the increase, resulting in an upward trend for the system’s total power consumption, thus leading to an increase in system emissions. The influence of the rising *W*_*net*_ surpasses that of system emissions. Therefore, the *EIL* of DL-KC shows a decreasing trend with the *P*_1_.

**Fig 5:**
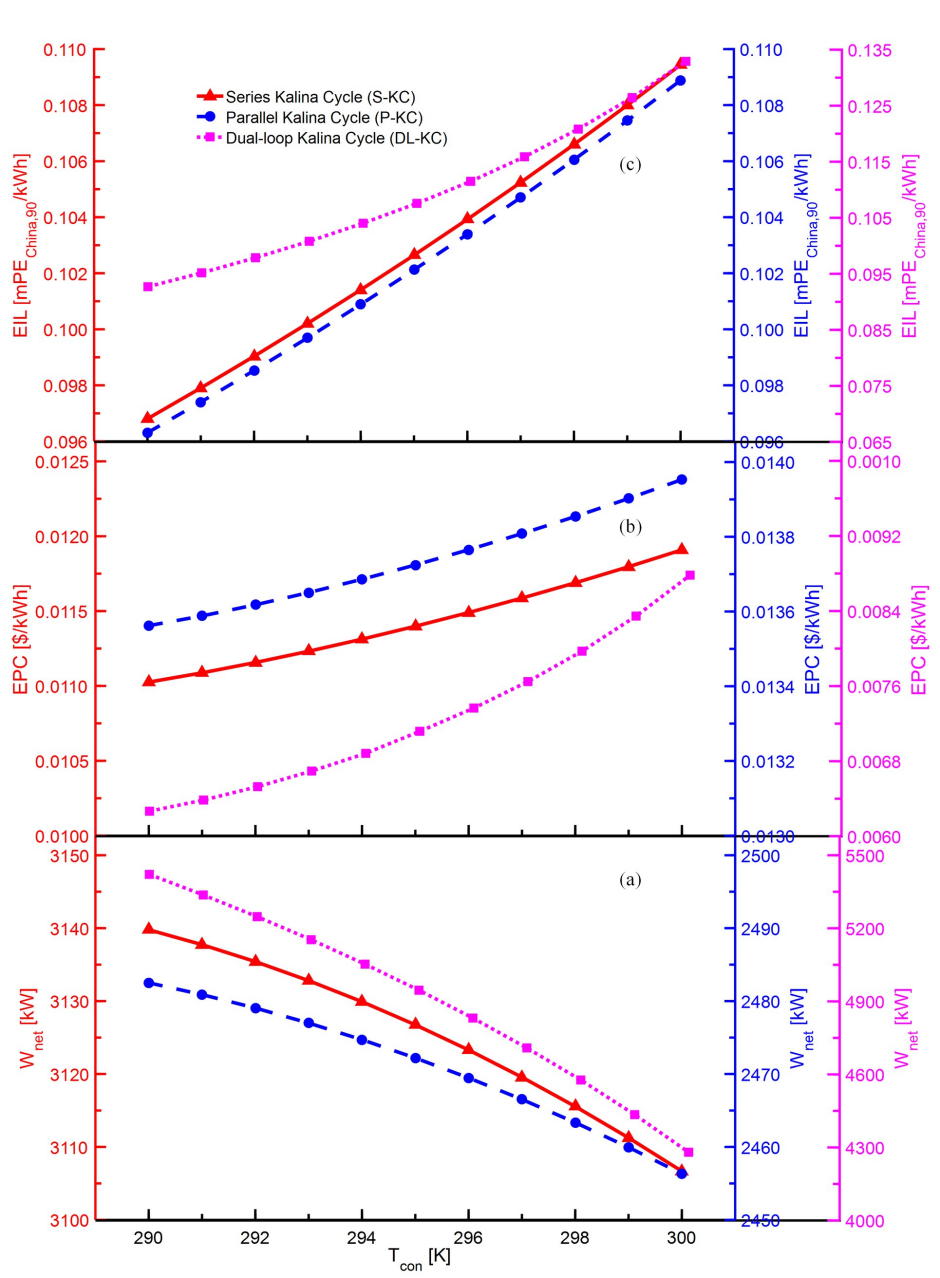
Performance variations of S-KC, P-KC and DL-KC with *T*_*con*_: (a) *W*_*net*_, (b) *EPC* and (c) *EIL*.

The variations of *W*_*net*_,*EPC* and *EIL* with *T*_*con*_ for S-KC, P-KC and DL-KC are shown in Fig 5. As shown in Fig 5(A), it can be seen that *T*_*con*_ has a negative influence on *W*_*net*_ for S-KC, P-KC and DL-KC. For the three systems, as *T*_*con*_ increases, the turbine’s inlet parameter remains unchanged, and its outlet’s enthalpy increases, thus leading to a drop in enthalpy difference, which brings a decrease in the power generation for the turbine. Meanwhile, owing to the increase in mass flow of ammonia-water mixture, the power consumption of the pump displays an increasing trend. The combined effects of the reducing turbine power production and increasing pump power consumption cause a decline in *W*_*net*_. Similar conclusion has also been obtained in the literature [56]. It is noted that the variation in *W*_*net*_ of S-KC, P-KC is relatively slight, while that of DL-KC is more obvious. As displayed in Fig 5(B), for S-KC, P-KC and DL-KC, the *EPC* grows with the increasing *T*_*con*_. When the heat energy absorbed from the heat source keeps constant, the increasing *T*_*con*_ enables lower mean temperature difference in the evaporator, inducing a growth in the heat transfer area, which causes an increment of system investment cost. The integrated effects of the increasing system investment cost and the falling *W*_*net*_ lead to a rising in the *EPC*. As observed in the research [56], there is a non-linear relationship between condenser’s temperature and unit cost. This discrepancy can be explained that in their study, the total cost has always shown a decreasing trend and plays a major role at the beginning of the condensation temperature, while the generated power has more effect with the condensation temperature further increasing. As exhibited in Fig 5(C), for the given *T*_*con*_, the *EIL* of S-KC, P-KC and DL-KC exhibit the increasing trends. The reason can be explained that the increasing *T*_*con*_ ensures more mass flow in the pump, leading to an increase in pump’s power consumption, thus raising the system emissions. The *EIL* of all three cycles grow as a consequence of the increase in system emissions and the falling *W*_*net*_.

**Fig 6:**
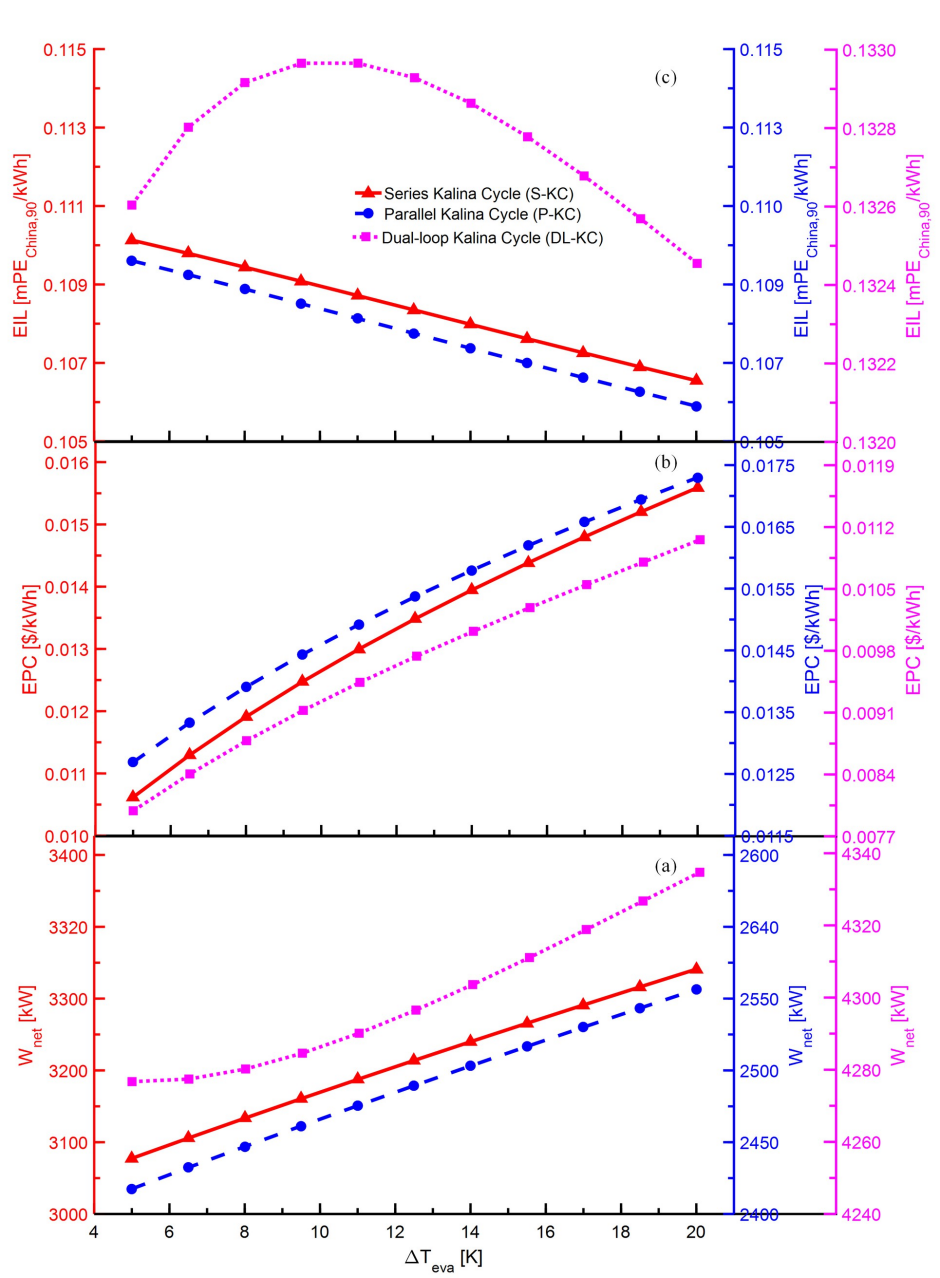
Performance variations of S-KC, P-KC and DL-KC with *ΔT*_*eva*_: (a) *W*_*net*_, (b) *EPC* and (c) *EIL*.

The variations of *W*_*net*_,*EPC* and *EIL* with Δ*T*_*eva*_ for S-KC, P-KC and DL-KC are shown in Fig 6. As indicated in Fig 6(A), when the *ΔT*_*eva*_ increases from 5 K to 20 K, the *W*_*net*_ raises from 3062 kW to 3273 kW for S-KC, from 2421 kW to 2588 kW for P-KC, and from 4277 kW to 4335 kW for DL-KC. For S-KC and P-KC, as the *ΔT*_*eva*_ augments, the *W*_*net*_ show the growing trend owing to the increasing ammonia-rich vapor and enthalpy difference in the turbine. For DL-KC, the production power of HP turbine grows because of the increasing ammonia-rich vapor and enthalpy difference in the HP loop, while that of LP turbine descends owing to the declining ammonia-rich vapor in the LP loop. The influence of HP turbine outweighs that of LP turbine, thus leading to the enhancement of *W*_*net*_. As shown in Fig 6(B), with *ΔT*_*eva*_ increases, there are the trends for *EPC* to become higher for three systems. With the increasing *ΔT*_*eva*_, the enthalpy at the outlet of the evaporator increases, causing a growth in the enthalpy difference, which leads to a raise in heat obtained by the evaporator. As a result, the heat transfer area shows an increasing trend, thus increasing the system investment cost. As the influence of the raising system investment cost exceeds that of the growing *W*_*net*_, the *EPC* increases. As displayed in Fig 6(C), the *EIL* of S-KC and P-KC decline with the increasing *ΔT*_*eva*_, while that of DL-KC raises first and then reduces. An increase in *ΔT*_*eva*_ enables larger heat exchange area in the evaporator, which causes an increase in the system’s total emissions. For S-KC and P-KC, the *W*_*net*_ raise faster than that of system emissions, causing the decline in *EIL*. For DL-KC, the growing system emissions have the dominant influence, which induce the rise in *EIL* at first. With the further increment of *ΔT*_*eva*_, the growing *W*_*net*_ becomes gradually obvious, and consequently the *EIL* declines later.

**Fig 7:**
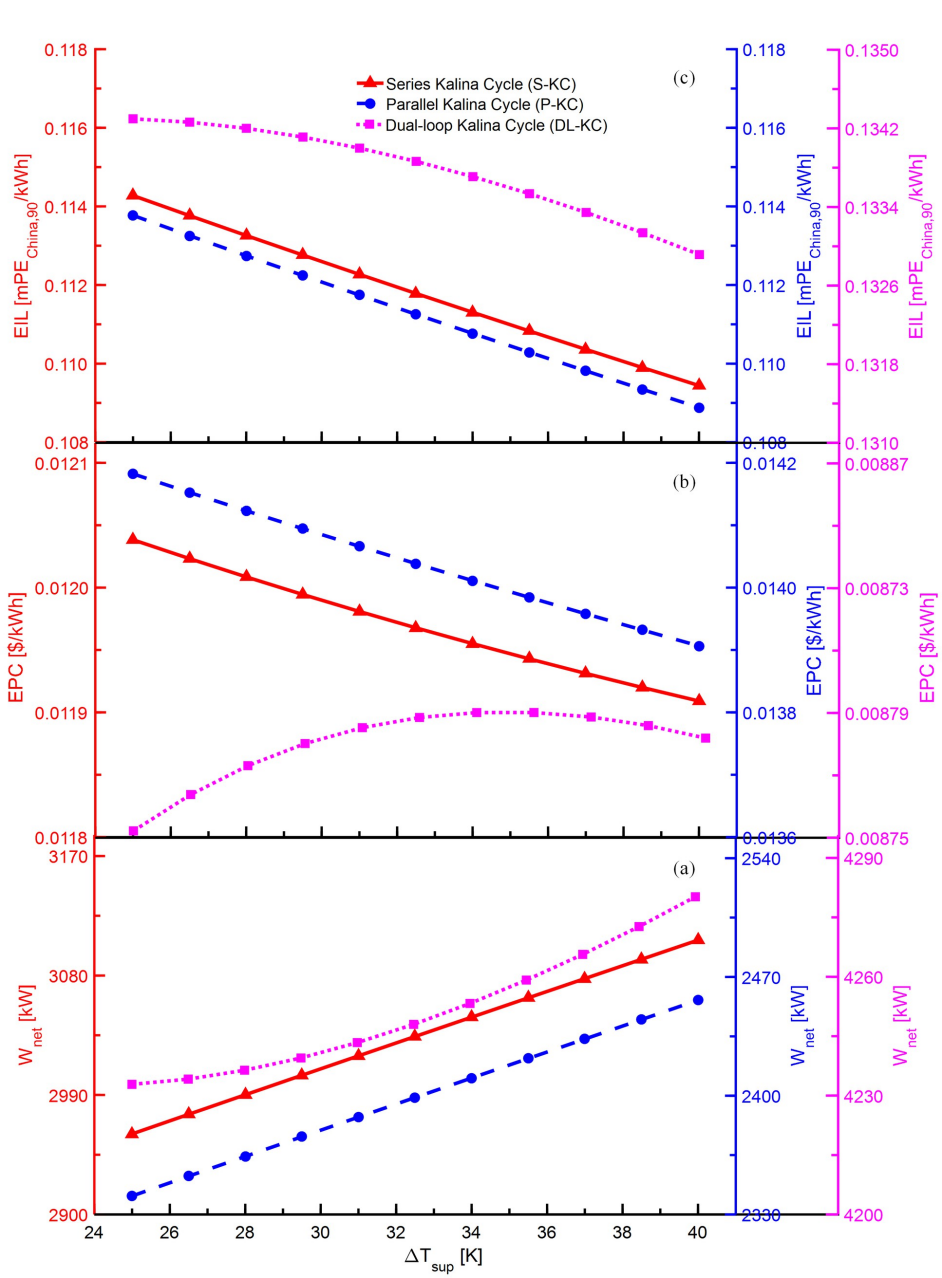
Performance variations of S-KC, P-KC and DL-KC with *ΔT*_*sup*_: (a) *W*_*net*_, (b) *EPC* and (c) *EIL*.

The variations of *W*_*net*_,*EPC* and *EIL* with *ΔT*_*sup*_ for S-KC, P-KC and DL-KC are shown in Fig 7. As shown in Fig 7(A), the increasing *ΔT*_*sup*_ results in the ascent of *W*_*net*_ for the KCs. Despite the increasing *ΔT*_*sup*_ leads to the rise in enthalpy at both the inlet and outlet of the turbine, the inlet enthalpy variation is more apparent, thus causing an increase in enthalpy difference. For S-KC and P-KC, the ammonia-rich vapor’s mass flow maintains constant, thus the increasing enthalpy difference in turbine raise its production power. When the pump’s power consumption keeps unchanged, the turbine’s production power increases, hence the *W*_*net*_ grows. For DL-KC, despite the LP turbine’s power generation declines due to the decrease in ammonia-rich vapor’s mass flow, the increase in the HP turbine has a greater impact, so the *W*_*net*_ shows an increasing tendency with the *ΔT*_*sup*_. As shown in Fig 7(B), the *EPC* of S-KC and P-KC decline, and the *EPC* of DL-KC increases first and then decreases when the *ΔT*_*sup*_ increases. With the increasing *ΔT*_*sup*_, the enthalpy of the superheater outlet keeps rising, while that of inlet remains at a fixed value, causing an increase in enthalpy difference of the superheater, which increases the heat exchange amount between the superheater and heat source. Meanwhile, the mean heat transfer temperature difference in superheat zone declines. The influences of the increasing heat exchange amount and the declining mean heat transfer temperature difference lead to a growth in the heat transfer area of the superheater, thus raising system investment cost. For S-KC and P-KC, the *W*_*net*_ increase faster than that of system investment cost, inducing the decrease in *EPC*. For DL-KC, the growth of system investment cost is dominant at first, and the *EPC* increases, while the growth of *W*_*net*_ is superior later, and the *EPC* declines. As shown in Fig 7(C), the *EIL* display the descending trends with *ΔT*_*sup*_ for the three systems. The system’s increasing heat transfer area enables higher total emissions. The *W*_*net*_ raise faster than that of system emissions, leading to the reductions in *EIL*.

**Fig 8:**
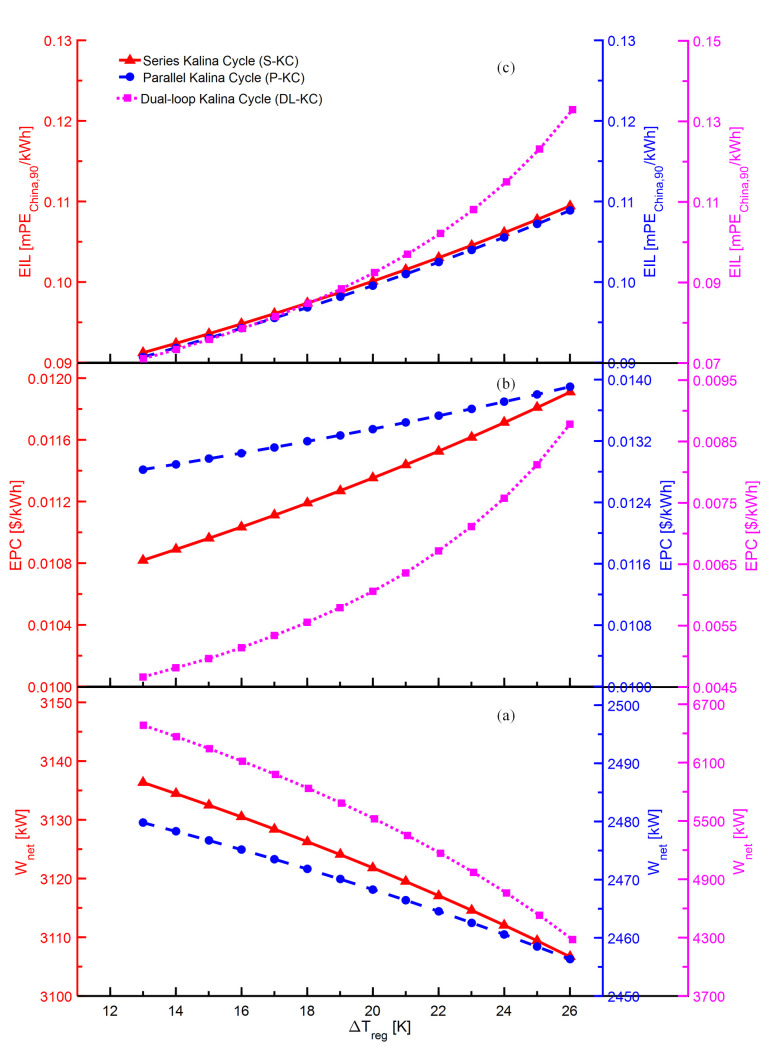
Performance variations of S-KC, P-KC and DL-KC with Δ*T*_*reg*_: (a) *W*_*net*_, (b) *EPC* and (c) *EIL*.

The variations of *W*_*net*_,*EPC* and *EIL* with Δ*T*_*reg*_ for S-KC, P-KC and DL-KC are shown in Fig 8. As exhibited in Fig 8(A), it can be seen that the Δ*T*_*reg*_ presents a negative influence on the *W*_*net*_ for S-KC, P-KC and DL-KC. The increase of Δ*T*_*reg*_ ensures the mass flow of pump to increase, causing a growth in the pump’s power consumption. Meanwhile, the mass flow of turbine decreases with the increasing Δ*T*_*reg*_, which reduces the power produced by the turbine. Therefore, the *W*_*net*_ declines gradually due to the decreasing power production in the turbine and the increasing power consumption in the pump. As displayed in Fig 8(B), the *EPC* of three systems increase with the Δ*T*_*reg*_. The increasing Δ*T*_*reg*_ enables the regenerator more heat exchange amount and lower mean heat transfer temperature difference, and thus its heat transfer area increases. Besides, for the given Δ*T*_*reg*_, there is also an increasing trend for the heat exchange areas of the evaporator unit and condenser. Therefore, the total heat exchange area of the system increases, which enables an increase in system cost. The comprehensive influences of the growing system cost and the descending *W*_*net*_ cause the increase in *EPC*. As shown in Fig 8(C), the Δ*T*_*reg*_ presents a positive impact on *EIL* for S-KC, P-KC and DL-KC. The increasing Δ*T*_*reg*_ causes an increment in the system’s total heat exchange area, then eventuating an augment in emissions. Therefore, the *EIL* exhibits an increasing trend due to the growing emissions and the declining *W*_*net*_.

**Fig 9:**
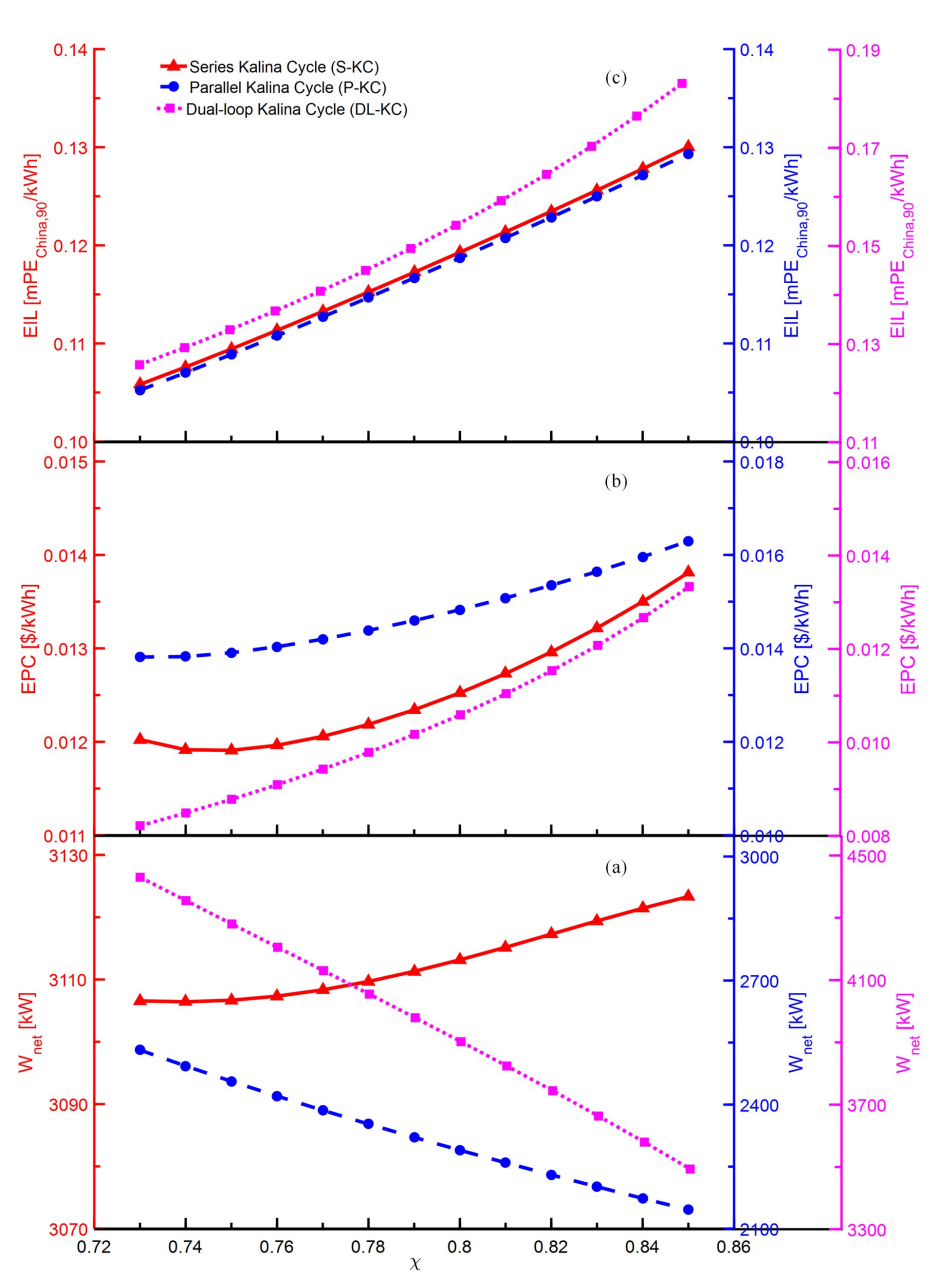
Performance variations of S-KC, P-KC and DL-KC with *x*: (a) *W*_*net*_, (b) *EPC* and (c) *EIL*.

The variations of *W*_*net*_,*EPC* and *EIL* with *x* for S-KC, P-KC and DL-KC are shown in Fig 9. As shown in Fig 9(A), for the given *x*, the *W*_*net*_ of S-KC almost maintains increasing while that of P-KC and DL-KC keep decreasing. The main explanation behind this fact is that the increasing *x* raises the mass flow of the system’s basic ammonia-water mixture, which augments the power consumption in the pump. Meanwhile, the power generation in the turbine of S-KC grows owing to the increase in mass flow of ammonia-rich vapor, while that of P-KC and DL-KC decrease because of the dropping mass flow of working fluid in the turbine. For S-KC, the increase in turbine’s power production nearly exceeds the pump’s power consumption. Therefore, the *W*_*net*_ shows an almost enhancing trend. For P-KC and DL-KC, the growing power consumption of the pump and the declining power production of the turbine cause the reducing *W*_*net*_. As revealed in Fig 9(B), as the *x* augments, the *EPC* of S-KC decreases for *x*<0.75 and increases for *x*>0.75, while the *EPC* of P-KC and DL-KC maintain raising. The evaporator unit is the key component that affects the system cost. The increasing *x* boosts both the heat exchange amount and mean heat transfer temperature difference in the evaporation process for three thermodynamic cycles. For S-KC, when the *x* is lower than 0.75, the increase of the mean heat transfer temperature difference in the evaporator has a greater impact than that of the heat exchange amount, inducing a descent in the heat transfer area, then leading to a reduction in the total component cost, and the *EPC* declines first. When the *x* is higher than 0.75, heat exchange amount of the evaporator shows a faster growth, causing a growth in the heat transfer area, so leading to a raise in the total component cost, and subsequently the *EPC* increases. For P-KC and DL-KC, the heat exchange amount in the evaporator always increases faster than that of mean heat transfer temperature difference, causing the growth in the heat transfer area, hence increasing the system cost, and their *EPC* show the increasing trends. The reference [35] also drawn a similar conclusion to this case. Generally, when the *x* approaches 1, its thermodynamic performance is closer to pure ammonia, and the corresponding phase transition temperature slip decreases, gradually losing the superiority of variable temperature phase transition. This means that the heat transfer area of the evaporator increases significantly, hence the system investment raises, and the economic performance gets worse. As shown in Fig 9(C), with the increasing *x*, the *EIL* increases from 0.106 mPE_China,90_/kWh to 0.130 mPE_China,90_/kWh for S-KC, from 0.105 mPE_China,90_/kWh to 0.129 mPE_China,90_/kWh for P-KC, and from 0.126 mPE_China,90_/kWh to 0.183 mPE_China,90_/kWh for DL-KC. The explanation could be that the increasing *x* enables higher power consumption of the pump and larger heat transfer area of the evaporator unit, which both increase the system total emissions. The effect of the growing system emissions exceeds that of the increasing *W*_*net*_, and the *EIL* exhibits an increasing trend for S-KC, while the comprehensive effects of the raising system emissions and the reducing *W*_*net*_ result in the increase in *EIL* for P-KC and DL-KC.

### 3.2. Optimization results

#### 3.2.1. Optimization results

Three optimization design scenarios are carried out for S-KC, P-KC and DL-KC. The single-objective optimization scenario refers to thermodynamic optimal design (TOD), the bi-objective optimization scenario involves thermodynamic and economic optimal design (TEOD), and the tri-objective optimization scenario considers thermodynamic, economic and environmental optimal design (TEEOD).

**Fig 10:**
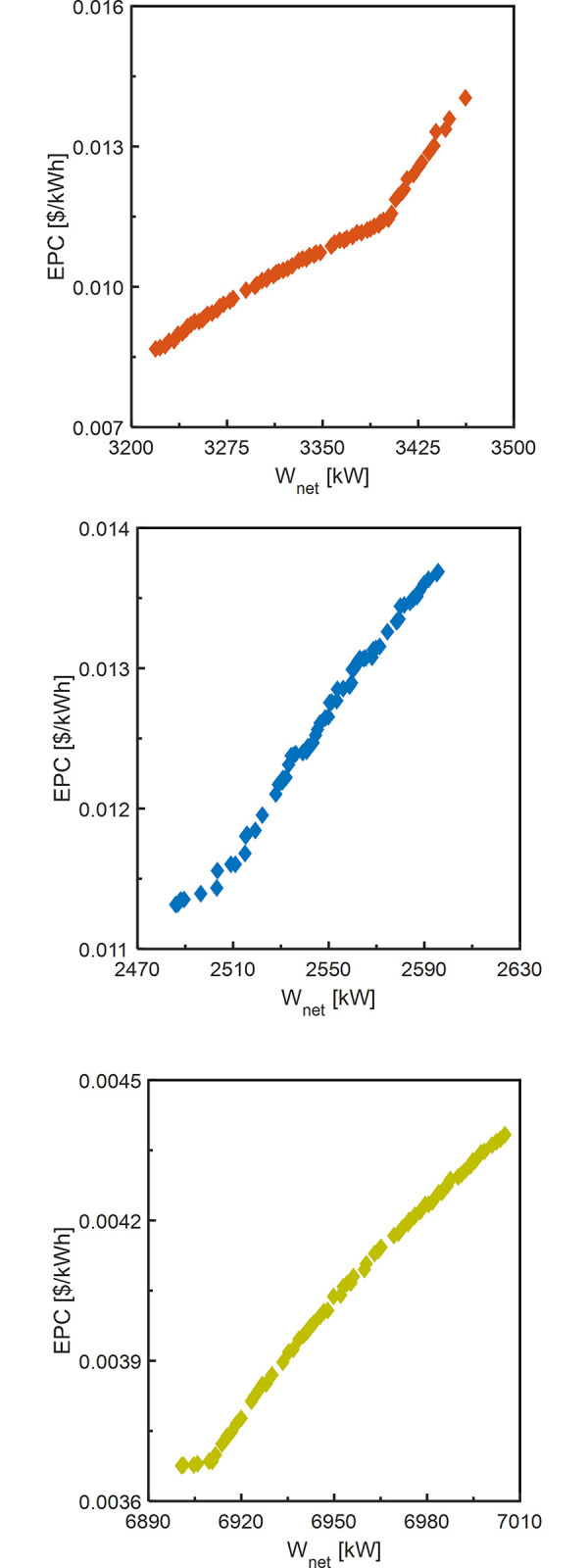
The thermodynamic cycle’s Pareto frontier under TEOD scenario: (a) S-KC, (b) P-KC and (c) DL-KC.

**Fig 11:**
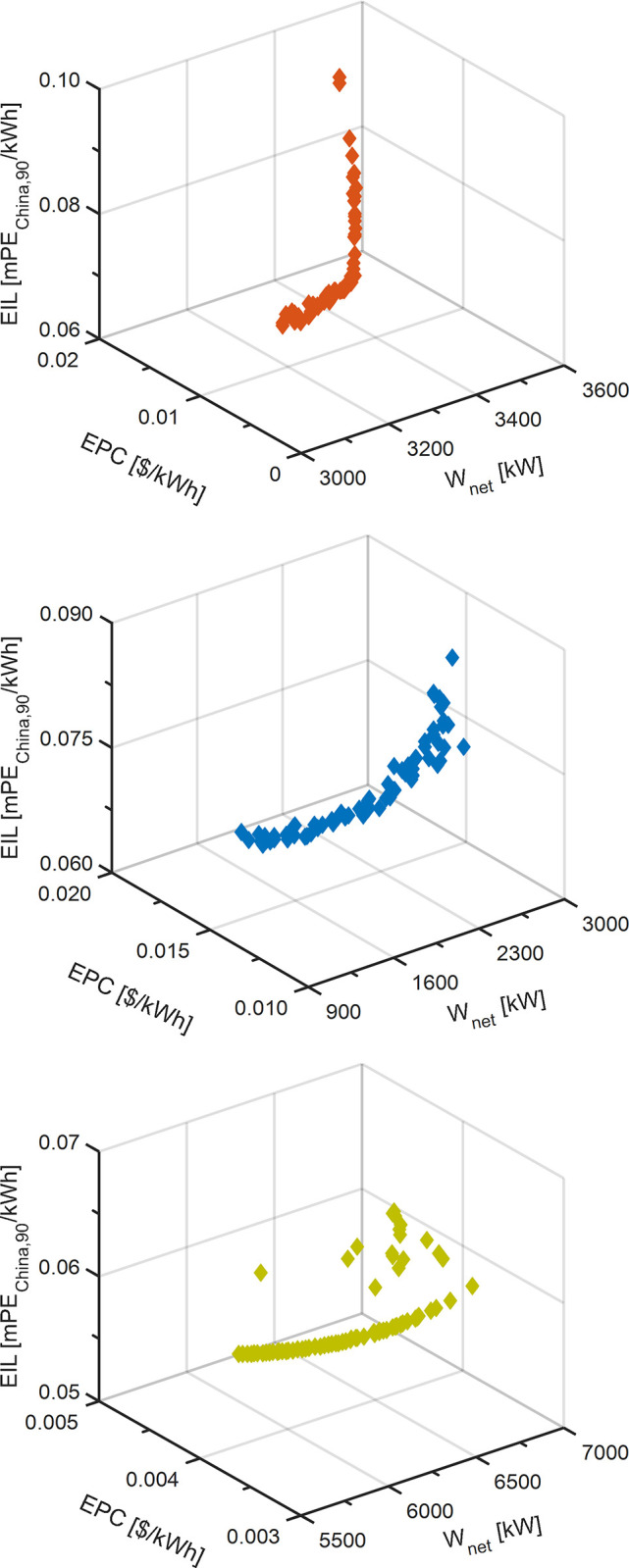
The thermodynamic cycle’s 3-D series of Pareto frontier under TEEOD scenario: (a) S-KC, (b) P-KC and (c) DL-KC.

For TOD scenario, the optimization results of S-KC, P-KC and DL-KC are listed in Table 7. For TEOD and TEEOD scenarios, they involve multi-objective optimization, the results of which are a series of optimal solutions. Fig 10 displays the Pareto frontier solution under TEOD scenario for S-KC, P-KC and DL-KC, and the Pareto-optimal solution and the corresponding operating parameters are presented in Table 8. Fig 11 exhibits the Pareto frontier under TEEOD scenario for S-KC, P-KC and DL-KC, and the Pareto-optimal solution and the corresponding operating parameters are listed in Table 9.

**Table 7:** Optimization results for S-KC, P-KC and DL-KC under TOD scenario.

System	Optimum objective	Objective value		Optimum variable					
	W_net_ (kW)	EPC ($/kWh)	EIL (mPE_China,90/_kWh)	P_1_ (kPa)	T_con_ (K)	ΔT_eva_ (K)	ΔT_sup_ (K)	ΔT_reg_ (K)	*x*
S-KC	3473	0.0175	0.1055	3516	290.01	20.00	40.00	26.00	0.849
P-KC	2692	0.0149	0.0788	5500	290.01	20.00	40.00	13.00	0.730
DL-KC	7166	0.0048	0.0597	5500	290.00	20.00	39.99	13.00	0.732

**Table 8:** Optimization results for S-KC, P-KC and DL-KC under TEOD scenario.

System	Optimum objective		Objective value	Optimum variable					
	W_net_ (kW)	EPC ($/kWh)	EIL (mPE_China,90_/kWh)	P_1_ (kPa)	T_con_ (K)	ΔT_eva_ (K)	ΔT_sup_ (K)	ΔT_reg_ (K)	*x*
S-KC	3226	0.0087	0.0710	3699	290.70	5.48	39.63	13.38	0.731
P-KC	2504	0.0114	0.0818	5411	292.02	5.51	39.90	13.12	0.733
DL-KC	6904	0.0037	0.0599	5265	290.03	5.06	38.08	13.01	0.731

**Table 9:** Optimization results for S-KC, P-KC and DL-KC under TEEOD scenario.

System	Optimum objective			Optimum variable					
	W_net_ (kW)	EPC ($/kWh)	EIL (mPE_China,90_/kWh)	P_1_ (kPa)	T_con_ (K)	ΔT_eva_ (K)	ΔT_sup_ (K)	ΔT_reg_ (K)	*x*
S-KC	3223	0.0089	0.0705	3505	290.44	5.76	38.43	13.14	0.738
P-KC	2479	0.0125	0.0882	5369	291.70	7.68	37.96	19.69	0.735
DL-KC	6838	0.0036	0.0592	5058	290.00	5.01	25.00	13.00	0.730

#### 3.2.2. System performance comparisons of S-KC, P-KC and DL-KC under TOD, TEOD and TEEOD scenarios

**Fig 12:**
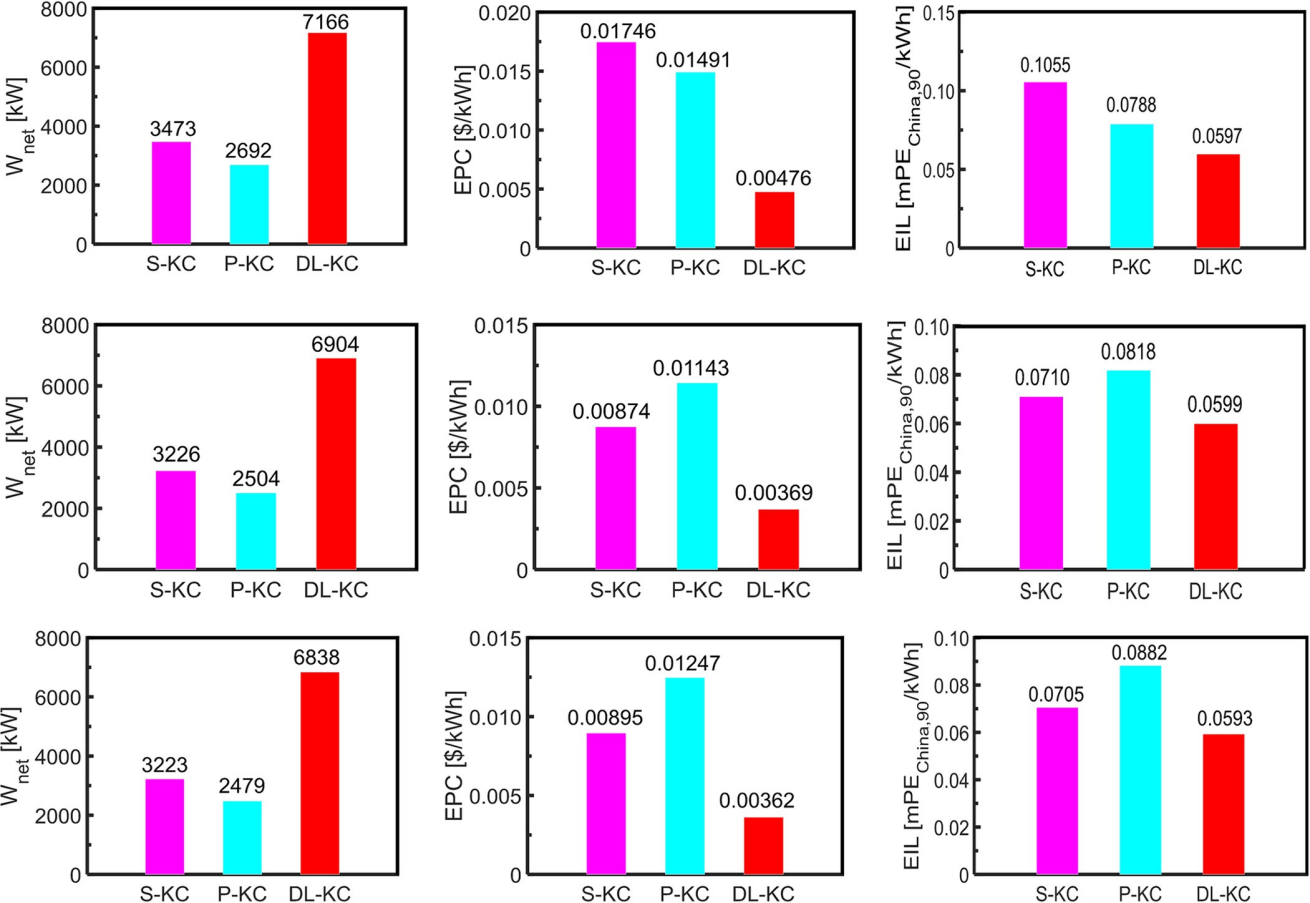
The *W*_*net*_,_EPC_ and *EIL* comparisons of S-KC, P-KC and DL-KC under three scenarios: (a) TOD, (b) TEOD.

Fig 12 exhibits the system performance comparisons including *W*_*net*_,*EPC* and *EIL* for each thermodynamic cycle under TOD, TEOD and TEEOD scenarios.

and (c) TEEOD.

As shown in Fig 12(A), under TOD scenario, the DL-KC has the highest *W*_*net*_ of 7166 kW, the lowest *EPC* of 0.00476 $/kWh, and the minimum *EIL* of 0.0597 mPE_China,90_/kWh. Namely, DL-KC is the best selection under TOD optimal design scenario from the perspectives of the thermodynamics, economics and environment. The S-KC possesses the moderate result of the *W*_*net*_, and its value is 3473 kW, and reaches the highest value of *EPC* and *EIL*, and the corresponding results are 0.01746 $/kWh and 0.1055 mPE_China,90_/kWh. For P-KC, it exhibits the least ideal thermodynamic performance with the *W*_*net*_ of 2692 kW, and has the medium values for *EPC* of 0.01491 $/kWh and *EIL* of 0.0788 mPE_China,90_/kWh.

As shown in Fig 12(B), under TEOD scenario, the system with the best performances from the viewpoints of thermodynamics, economics and environment is DL-KC, followed by S-KC and P-KC. At this optimal scenario, DL-KC also gives the highest *W*_*net*_, the lowest *EPC* and the minimum *EIL*, with the corresponding values of 6904 kW, 0.00369 $/kWh and 0.0599 mPE_China,90_/kWh, while P-KC has lowest *W*_*net*_, the highest *EPC* and maximum *EIL*, with the corresponding results of 2504 kW, 0.01143 $/kWh and 0.0818 mPE_China,90_/kWh. In other words, DL-KC is also the best choice under the TEOD scenario for dual-level heat recovery in cement industry, while P-KC is the worst selection.

As shown in Fig 12(C), under TEEOD scenario, the comparisons among the three systems demonstrate that the power cycle with the best performances in terms of thermodynamics, economics and environment is DL-KC, followed by S-KC and P-KC (similar to the TEOD scenario). DL-KC produces more *W*_*net*_ than S-KC and P-KC with the corresponding values of 3615 kW and 4359 kW, owns lower *EPC* than S-KC and P-KC with the corresponding values of 0.00533 $/kWh and 0.00885 $/kWh, has less *EIL* than S-KC and P-KC with the corresponding values of 0.0113 mPE_China,90_/kWh and 0.0290 mPE_China,90_/kWh. At this optimal scenario, DL-KC is also the most suitable system from thermodynamics, economics and environment perspectives for the cement’s WHR, while P-KC is the least attractive choice.

#### 3.2.3. Component performance comparisons of S-KC, P-KC and DL-KC under TOD, TEOD and TEEOD scenarios

**Fig 13:**
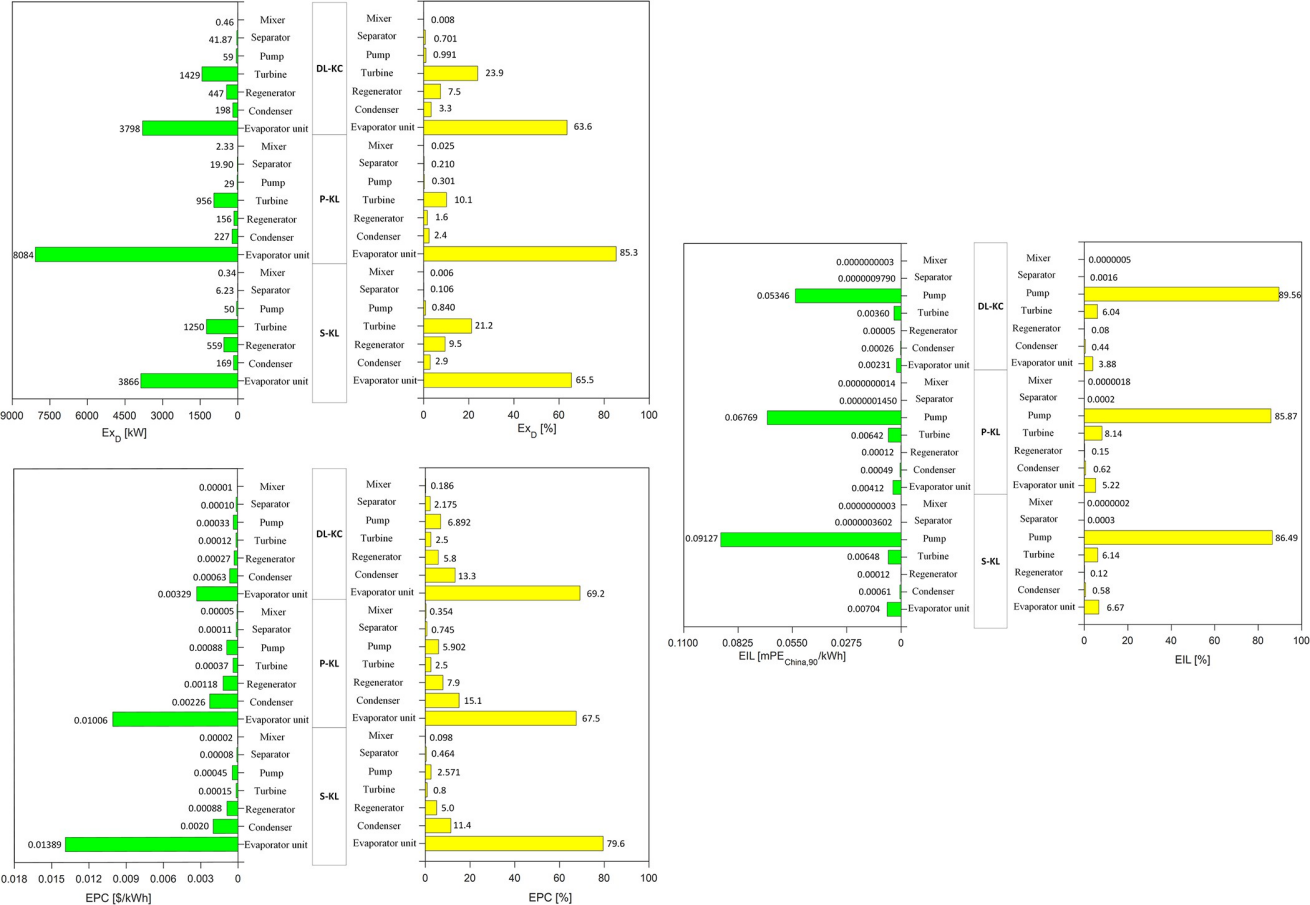
Analysis of ***Ex***_***D***_**,*EPC* and *EIL*** of each component for S-KC, P-KC and DL-KC under TOD scenario.

Fig 13 shows the distributions of *Ex*_*D*_,*EPC* and *EIL* of each component for S-KC, P-KC and DL-KC under TOD scenario. Regarding *Ex*_*D*_, it is discovered that the evaporator unit has the highest values of 3866 kW, 8084 kW and 3798 kW for S-KC, P-KC and DL-KC, accounting for 65.5%, 85.3% and 63.6%, respectively. This conclusion is also consistent with the existing researches [57–59]. The *Ex*_*D*_ produced from the evaporator unit is chiefly caused by the large heat transfer temperature difference during the heat exchange process, thus improving evaporator unit’s efficiency is an effective method to reduce this *Ex*_*D*_. The proportion of the turbine is the second highest, corresponding to 21.2%, 10.1% and 23.9% for the KCs. For the rest components, the contributions are not significant, usually less than 10%. With respect to *EPC*, the evaporator unit also acts a crucial role in the total *EPC*. The *EPC* of S-KC is 0.01389 $/kWh, contributing to 79.6%, P-KC is 0.01006 $/kWh, contributing to 67.5%, and DL-KC is 0.00329 $/kWh, contributing to 69.2%. The complicated structure of the evaporation unit consisting of an economizer, evaporator and superheater enables the highest heat transfer area, hence this component occupies the largest share of the system. It is noted that, the studies [57, 60] indicated that the majority component cost of KC associated with the turbine, while the literature [61] pointed out the evaporator also accounted for the highest proportion. Different conclusions in existing researches could be due to the discrepancy of KC’s configuration and operating condition. In present paper, the condenser occupies the second proportion and possesses 11.430%, 15.129% and 13.318% for S-KC, P-KC and DL-KC. The remaining compositions are less apparent. Regarding *EIL*, for S-KC, P-KC and DL-KC, the pump is the major contribution, with the values of 0.09127 mPE_China,90_/kW, 0.06769 mPE_China,90_/kW and 0.05346 mPE_China,90_/kW, and the proportions are 86.49%, 85.87% and 89.57%. The ratios occupying by evaporator unit range from 6.67% to 3.88% while that of turbine are 8.14% to 6.04%. In other words, the pump is the decisive component that affects the *EIL*. The fact could be that, in the operation phase, a large amount of NO_x_ and CH_4_ have generated when the pump consumes electric energy. For all thermodynamic cycles run under TOD scenario, the evaporator unit has the maximum *Ex*_*D*_ and *EPC*, while the pump exhibits the highest *EIL*. Therefore, it is necessary to pay more attention to the evaporator unit for reducing system’s *Ex*_*D*_ and *EPC*, and show more concern for pump to decrease *EIL*.

**Fig 14:**
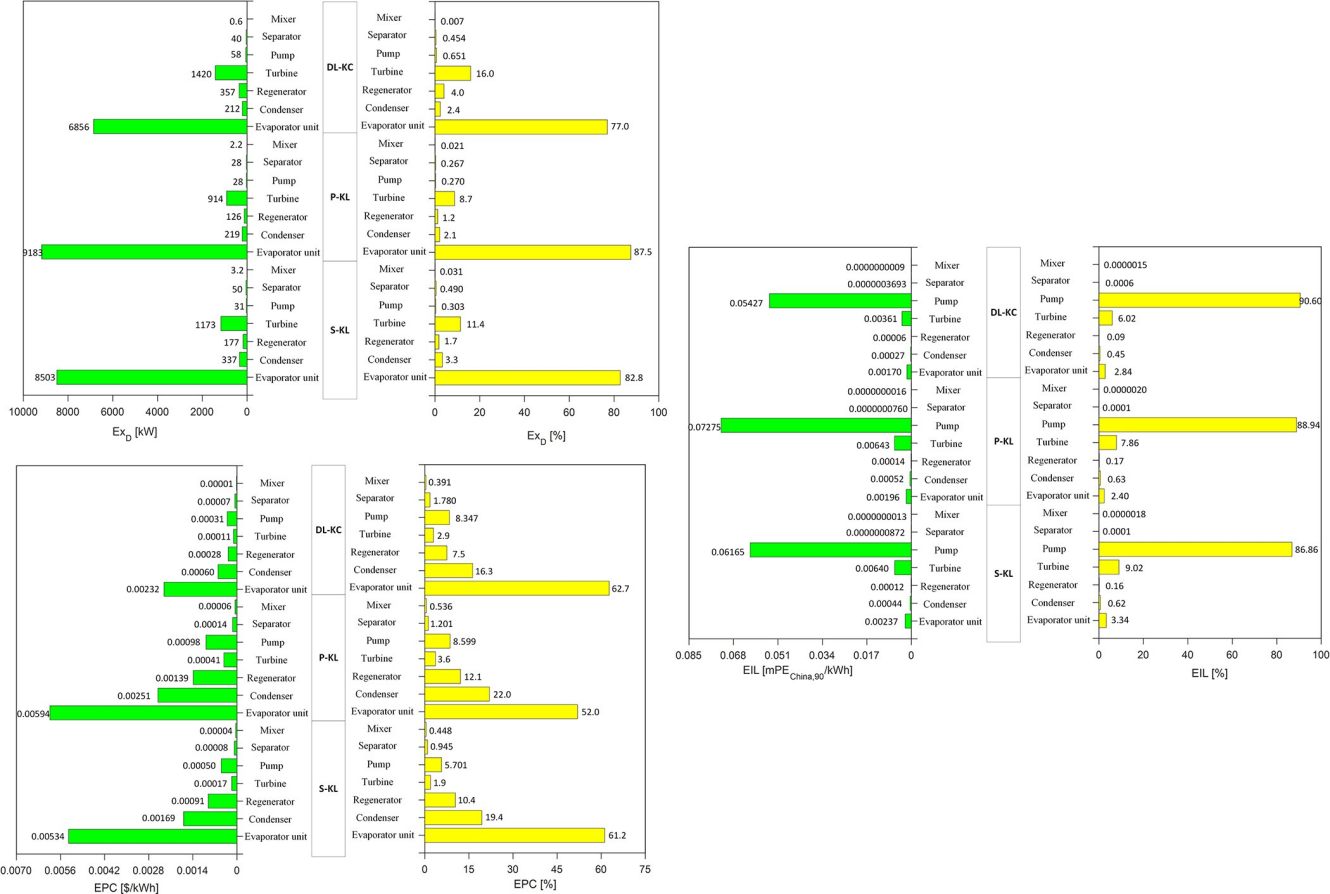
Analysis of ***Ex***_***D***_**,*EPC* and *EIL*** of each component for S-KC, P-KC and DL-KC under TEOD scenario.

Fig 14 shows the distributions of *Ex*_*D*_,*EPC* and *EIL* of each component for S-KC, P-KC and DL-KC under TEOD scenario. For *Ex*_*D*_, it could also be found that the evaporator unit is the key source with proportions of 82.8%, 87.5% and 77.0% for S-KC, P-KC and DL-KC, and then followed by turbine accounting for 11.4%, 8.7% and 16.0%. The components of S-KC, P-KC and DL-KC that give the higher proportions for *EPC* are evaporator unit and condenser. The proportions reach to 61.2%, 52.0% and 62.7% for evaporator unit, and 19.4%, 22.0% and 16.3% for condenser. In terms of *EIL*, for the three systems, the contribution of pump is also dominant, occupying 86.86%, 88.94% and 90.60%, and the turbine makes the second contribution, achieving 9.02%, 7.86% and 6.02%. At this case, similar to TOD scenario, the evaporator unit has the greatest impact on *Ex*_*D*_ and *EPC*, and the pump has the maximum value of *EIL*.

**Fig 15:**
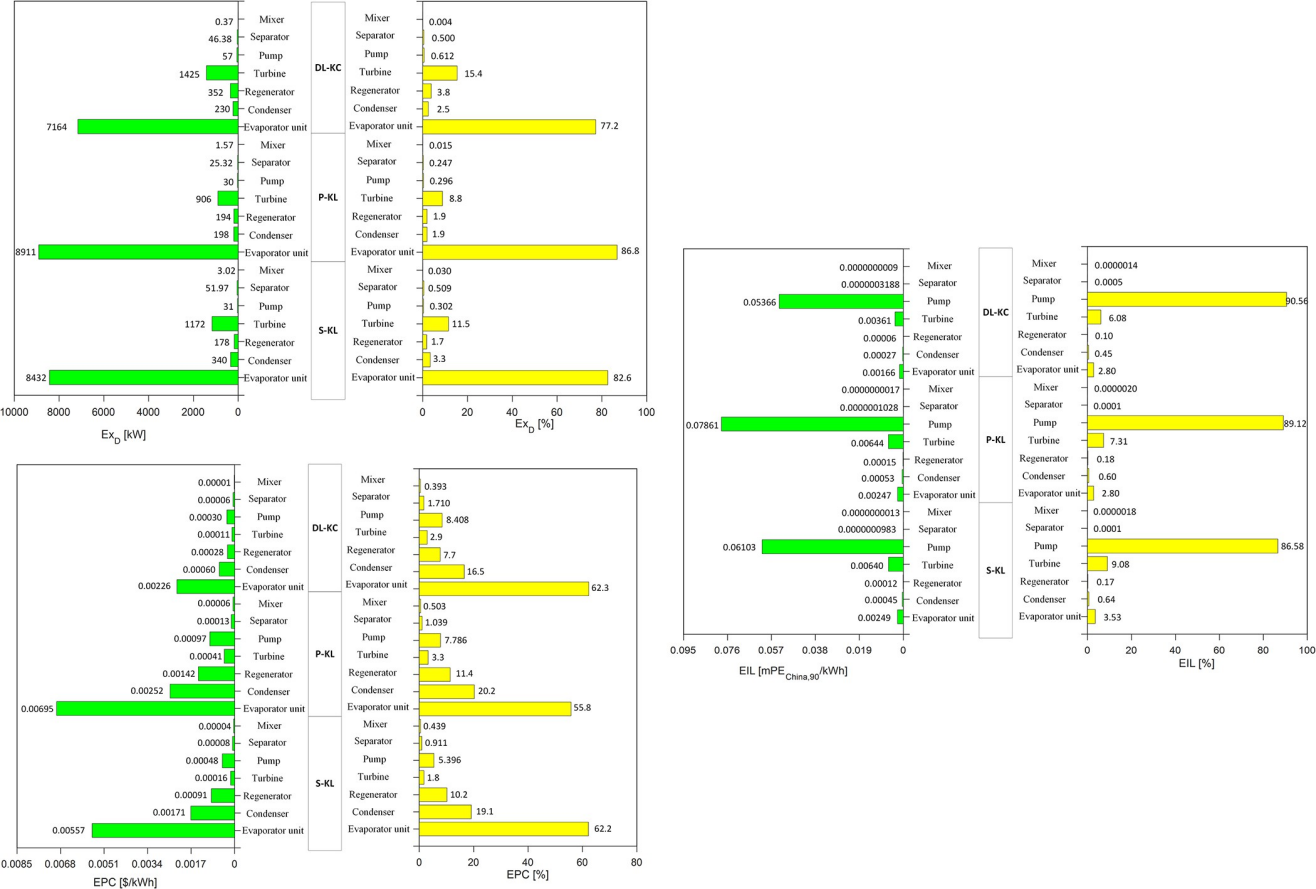
Analysis of *Ex*_*D*_,*EPC* and *EIL* of each component for S-KC, P-KC and DL-KC under TEEOD scenario.

Fig 15 shows the distributions of *Ex*_*D*_,*EPC* and *EIL* of each component for S-KC, P-KC and DL-KC under TEEOD scenario. From the *Ex*_*D*_ perspective, it can be seen that, the evaporator unit also exhibits the highest contribution, which is more than 77% for all thermodynamic cycles. The second *Ex*_*D*_ belongs to the turbine ranging from 8.8% to 15.4%. With respect to economic performance, the comparison results show that evaporator unit displays the highest *EPC* with the proportions of 55.8%-62.3% for the three cycles. The *EPC* is also mainly caused by condenser, and the scopes of which range from 16.5% to 20.2%. Regarding the environmental performance, the pump also possesses the maximum *EIL*, with the proportions of 86.58%-90.56% for the three systems. Although the turbine has the second impact on *EIL*, its proportion is less than 10%. At the TEEOD mode, similar to TOD and TEOD scenarios, the evaporator unit is also the key component contributing to *Ex*_*D*_ and *EPC*, while the pump is the dominant source for *EIL*.

## Section 4: Conclusions

Aiming to improve energy utilization efficiency in cement industry, the novel KCs including S-KC, P-KC and DL-KC have been designed to simultaneously recover exhaust gas from suspension preheater and hot air from clinker cooler for the first time. To perform system investigation, thermodynamic, economic, and environmental models are established in the MATLAB linked with REFPROP. For the proposed cycles, the influences of six leading parameters containing *P*_1_,*T*_*con*_,*ΔT*_*eva*_,*ΔT*_*sup*_,Δ*T*_*reg*_ and *x* on the *W*_*net*_,*EPC* and *EIL* are analyzed. Meanwhile, in terms of *W*_*net*_,*EPC* and *EIL*, the performances of systems and components are discussed and compared under TOD, TEOD and TEEOD optimal design scenario. The remark findings are summarized as follows:

(1) The impact of the operation parameters on the performance of the three systems is not consistent. However, the results show that for both S-KC, P-KC and DL-KC, higher *W*_*net*_ could be gained with decreasing *T*_*con*_,Δ*T*_*reg*_, and increasing *ΔT*_*eva*_,*ΔT*_*sup*_, lower *EPC* can be acquired with decreasing *T*_*con*_,*ΔT*_*eva*_ and Δ*T*_*reg*_, and less *EIL* can be attained with decreasing *T*_*con*_,Δ*T*_*reg*_,*x*, and increasing *ΔT*_*sup*_.

(2) For the studied cement industry, under TOD, TEOD and TEEOD scenarios, DL-KC is the best selection from the perspectives of thermodynamics, economics and environment, and it could generate *W*_*net*_ by 6838 kW-7166 kW, *EPC* by 0.00362$/kWh-0.00476 $/kWh and *EIL* by 0.0593 mPE_China,90_/kWh-0.0599 mPE_China,90_/kWh.

(3) For S-KC, P-KC and DL-KC, the evaporator unit is decisive in assessing exergy destruction and investment cost for WHR in cement industry, with *Ex*_*D*_ ratio ranges of 63.6%-85.3%, 77.0%-87.5% and 77.2%-86.8%, *EPC* ratio ranges of 67.5%-79.6%, 52.0%-62.7% and 55.8%-62.3%, implying that there is a lot of space for optimization of evaporator unit to enhance thermodynamic and economic performances, while the pump has the maximum influence on environmental performance, with *EIL* proportion scopes of 85.87%-89.56%, 86.86%-90.60% and 86.58%-90.56%, meaning more concern could be considered to pump for reducing environmental influence.

This study assesses thermodynamic, economic and environmental performance of the proposed KCs for dual-source heat recovery in cement industry, and the results could provide assistance for other investigators and industrial developers in component optimization design, and decisively adopting the desired system while fitting their scenarios. Nevertheless, in the current research, the performances of the double-source heat recovery systems are explored under design condition. In fact, as the operating conditions of the cement production change, the heat source characteristics such as temperature and mass flow also vary accordingly. Therefore, future research is still essential to investigate operating feature of the double-source KC under fluctuating heat source situations, explore the off-design and dynamic performances for matching the practical engineering application.

## Supporting information

S1 Nomenclature (DOCX)
