# Supplementary material for: Thermodynamics, economy and environment analyses and optimization of series, parallel, dual-loop Kalina cycles for double-source heat recovery in cement industry
Source: PLoS One. 2025 Feb 21;20(2):e0315972. doi: 10.1371/journal.pone.0315972 (PMC11844833; doi:10.1371/journal.pone.0315972)
Supplement: S1 Nomenclature — (DOCX) [file pone.0315972.s001.docx]

**Nomenclature**

$a$ heat-transfer coefficient (W/(m^2^·K)

$A$ heat transfer area (m^2^)

$C_{bm}$ component cost ($)

$C_{p}$ component benchmark cost ($)

$Ex$ exergy (kJ)

$F_{bm}$ cost factor

$f_{k}$ cost coefficient

$h$ enthalpy (kJ)

$h_{full-load}$ full-load operation time (h)

$i$ interest rate

$m$ mass (kg/s)

$P$ pressure (kP)

$Q$ energy (kJ); emission quantity (kg)

$s$ entropy(kJ)

$t$ thickness (m)

$T$ temperature (K)

$U$ total heat transfer coefficient (W/(m^2^·K))

$W$ power (kJ)

**Abbreviations**

AP acidification potential

CEPCI chemical engineering plant cost index

CRF capital recovery factor

DL-KC dual-loop Kalina Cycle

EF equivalent factor

EIL environment impact load

EP environment impact potential; eutrophication potential

EPC electricity production cost

ER environment impact potential of [region](http://dict.youdao.com/search?q=%5B%E6%95%B0%5D%20region%0D%0A&keyfrom=fanyi.smartResult)

GWP global warming potential

HP high-pressure

HTP human toxicity potential

KC Kalina cycle

LCA life cycle assessment

LP low-pressure

NEP data standardization

ORC organic Rankine cycle

P-KC parallel Kalina Cycle

POCP photochemical ozone creation potential

SAP soot and dust potential

SWP solid waste potential

S-KC series Kalina Cycle

TOD thermodynamic optimal design

TEOD thermodynamic and economic optimal design

TEEOD thermodynamic, economic and environmental optimal design

WF weighting factor

WHR Waste heat recovery

WP potential environment impact weight

**Greek letters**

$\chi$ ammonia concentration

$\lambda$ thermal conductivity (W/(m·K)

**Subscripts**

con condenser

cs cold side

D destruction

eva evaporator

hs hot side

in input

m mean

max maximum

min minimum

net net

out output

p pump

reg regenerator

sup superheat

tot total

turb turbine

0 dead state
